# Synthesis of Monothiacalix[4]arene Using the Fragment Condensation Approach

**DOI:** 10.3390/molecules30153145

**Published:** 2025-07-27

**Authors:** Daniel Kortus, Oliver Moravec, Hynek Varga, Michal Churý, Kamil Mamleev, Jan Čejka, Hana Dvořáková, Pavel Lhoták

**Affiliations:** 1 Department of Organic Chemistry, University of Chemistry and Technology Prague (UCTP), Technická 5, 166 28 Prague, Czech Republic; kortusd@vscht.cz (D.K.); moraveco@vscht.cz (O.M.); hynek.varga@email.cz (H.V.); churym@vscht.cz (M.C.); kamil.mamleev1997@gmail.com (K.M.); 2 Department of Solid State Chemistry, University of Chemistry and Technology Prague (UCTP), Technická 5, 166 28 Prague, Czech Republic; cejkaj@vscht.cz; 3 Laboratory of NMR Spectroscopy, University of Chemistry and Technology Prague (UCTP), Technická 5, 166 28 Prague, Czech Republic; dvorakoh@vscht.cz

**Keywords:** calixarene, thiacalixarene, bisphenols, synthesis, macrocyclization, fragment condensation, variable temperature NMR, conformation, *flip-flop* mechanism

## Abstract

The article describes a simple and scalable preparation of 2-monothiacalix[4]arene **7**, the simplest representative of the mixed-bridged (CH_2_ and S) calix[4]arenes. The synthesis is based on the condensation of linear building blocks (bisphenols), which are relatively readily available, and allows, depending on the conditions, the use of two alternative reaction routes that provide macrocycle **7** in high yield. The dynamic behavior of the basic macrocyclic skeleton was investigated using NMR spectroscopy at variable temperatures. High-temperature measurements showed that compound **7** undergoes a *cone*–*cone* equilibrium with activation free energy Δ*G*^#^ of the inversion process of 63 kJ·mol^−1^. Interestingly, the same barrier for the oxidized sulfone derivative **14** shows a value of 60 kJ·mol^−1^, indicating weakened hydrogen bonds at the lower rim of the calixarene. The same was also confirmed at low temperatures, when barriers to changing the direction of the cyclic hydrogen bond arrays (*flip-flop* mechanism) were determined (compare Δ*G*^#^ = 44 kJ·mol^−1^ for **7** vs. Δ*G*^#^ = 40 kJ·mol^−1^ for **14**).

## 1. Introduction

**Scheme 1:**
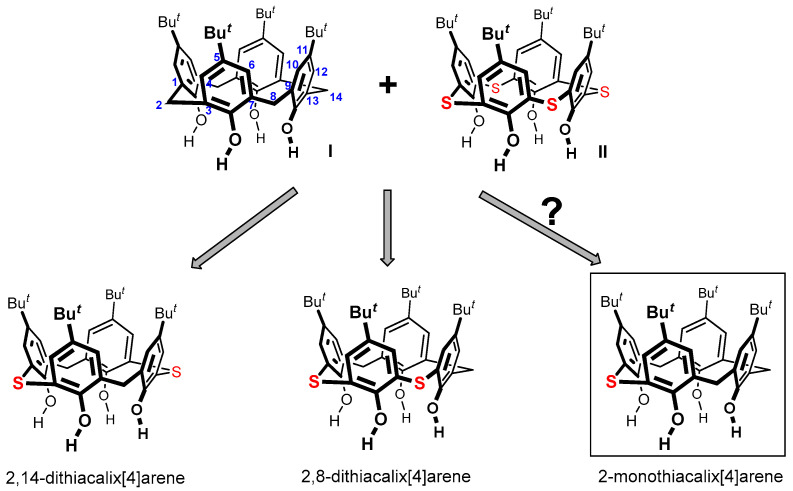
Crossbreeding of parent macrocycles **I** and **II** to form mixed bridge systems (the numbering of the basic skeleton is shown in blue).

Calix[n]arenes, well-known cyclic oligophenols with methylene bridges, have become an integral part of modern supramolecular chemistry, as they exhibit a number of interesting properties that can be exploited in the design and synthesis of sophisticated functional systems [1,2]. The possibility of almost any derivatization of the basic macrocyclic skeleton, together with the ability to fix the 3D shapes of the molecule in a pre-selected conformation, makes calix[4]arene **I** (Scheme 1) a perfect choice as a building block/molecular scaffold for the design of various receptors and self-assembly systems [3,4].

It is noteworthy that the introduction of sulfur atoms instead of methylene bridges [5], as is the case with the so-called thiacalix[4]arene **II** (Scheme 1), leads to many changes in the behavior of these systems, both from the perspective of chemical derivatization and in terms of conformational preferences or complexation behavior of the macrocycles [6,7,8].

Since both parent macrocycles have their own characteristic properties, specific for the given type of bridges (classical CH_2_ versus thia-derivatives), it turned out to be interesting to crossbreed parent molecules (Scheme 1) to create macrocycles with mixed bridges (CH_2_ and S). The successful synthesis of 2,8- and 2,14-dithiacalixarene [9,10] in multigram quantities has enabled a broader study of these systems. It was found that they exhibit unusual conformational preferences compared to the parent macrocycles [11,12], or that it is possible to achieve inherent chirality through a simple dialkylation of the lower rim (phenolic groups) [13].

**Scheme 2:**
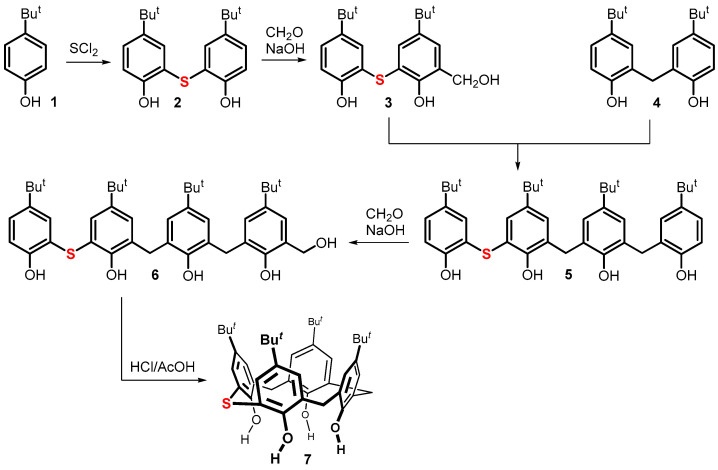
Synthesis of monothiacalix[4]arene **7** described in the literature.

While the chemistry of dithiacalix[4]arenes has been at least partially explored, a similar study of the monothia derivative (Scheme 1) is still lacking. The reason is the practical unavailability of this macrocycle, the only synthesis of which was reported three decades ago [14]. The synthesis of compound **7** is based (Scheme 2) on the preparation of sulfur bisphenol **2** by the reaction of the starting *p-tert*-butylphenol **1** with sulfur dichloride. This dimer is then converted to the corresponding hydroxymethylated derivative **3**, which is condensed with dimer **4** in the next step to form a linear tetramer **5** [15]. The hydroxymethylation of the tetramer provided compound **6**, which was finally cyclized under high dilution conditions to yield monothiacalix[4]arene **7**. Obviously, the reaction pathway contains several steps that are difficult to perform, especially the monohydroxymethylation reactions to form compounds **3** and **6**, which makes this stepwise synthetic procedure unsuitable for larger-scale synthesis.

Since almost nothing is known about the chemistry and general behavior of the target macrocycle **7** [16], a synthetic method that would provide the compound in acceptable yield with minimal effort would be desirable. During our ongoing research on the functionalization of the macrocyclic skeletons, we have found a simple and scalable preparation of 2-thiacalix[4]arene, the simplest representative of the mixed-bridge calixarene family. The synthesis is based on the condensation of linear building blocks (bisphenols), which are relatively easily available, and allows, depending on the circumstances, the use of two alternative reaction pathways providing the product **7** in high yield. This article describes the results of our synthetic efforts.

## 2. Results and Discussion

**Scheme 3:**
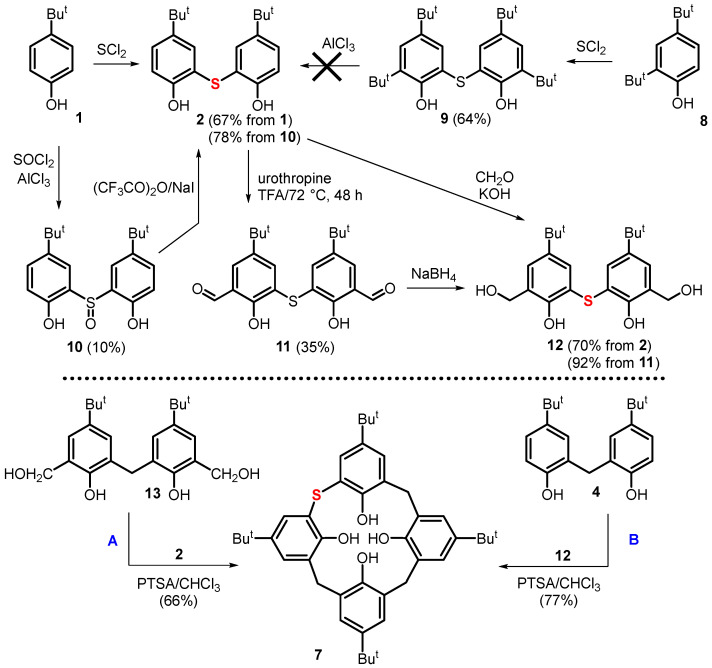
Synthesis of 2-thiacalix[4]arene **7**.

The synthesis of compound **7** is based on the preparation of two key linear building blocks **2** and **12** (Scheme 3). The reaction of SCI_2_ with an excess of the starting 4-*tert*-butylphenol **1** was carried out according to the literature procedure [9]. At a molar ratio of 1:7 (SCl_2_:**1**), a practically pure dimer is formed, which is, however, diluted with a large amount of unreacted starting phenol **1**. On the other hand, the use of a lower excess (1:2 or 1:3 SCl_2_:**1**) led to the formation of a relatively diverse product mixture with a significant presence of the trimer, which can be separated only by column chromatography. Unreacted phenol **1** can be removed by steam distillation, but this procedure is particularly lengthy and takes tens of hours for a 20 g amount. Moreover, it requires constant attention, as the condenser is often clogged with solidifying *tert*-butylphenol. Fortunately, it was found that this time-consuming procedure can be circumvented by a series of simple crystallizations of the crude reaction mixture from cyclohexane, which leads to the gradual removal of the unreacted starting material. This modification led to the isolation of bisphenol **2** in 67% yield.

Since bisphenol **2** is the cornerstone of the entire synthesis, we attempted its alternative synthesis. Based on the literature procedure [17], the commercially available 2,4-di-*tert*-butylphenol **8** was reacted with SCl_2_ in a 2:1 ratio in CH_2_Cl_2_ at 0 °C to give the corresponding dimer **9** in 64% yield. Unfortunately, all attempts to selectively remove the *tert*-butyl groups at the 2,2′ positions failed. Although a similar selective de-*tert*-butylation is described [18,19] for the corresponding CH_2_ analogue, suitable reaction conditions for the sulfur derivative could not be found.

Another problem associated with this reaction is the commercial unavailability of SCl_2_, which has completely disappeared from the offer of global suppliers of chemical specialties (apparently due to possible misuse for the production of chemical weapons). To avoid having to rely on our old supplies, we tried to get around this bottleneck by using thionyl chloride as an electrophilic reagent. The starting 4-*tert*-butylphenol **1** was reacted with AlCl_3_ and SOCl_2_ at room temperature in CH_2_Cl_2_ according to the literature procedure for *p*-cresol [20]. Although the analogous reaction of *p*-cresol provided the product in 65% yield, *tert*-butyl sulfoxide **10** was formed only in poor 10% yield. The sulfoxide **10** was then reduced using a trifluoroacetic anhydride/sodium iodide system [21] in acetone. The resulting sulfide **2** was isolated in 78% yield. However, due to the low yield of sulfoxide formation, this route is not very meaningful.

The bis(hydroxymethyl) derivative **12** can be prepared according to the literature procedure[9,15] by direct hydroxymethylation of **2** in 70% yield using KOH and an excess of formaldehyde (20 equiv.) in aqueous solution at 50 °C. To increase the variability of the building block synthesis, we attempted to introduce hydroxymethyl groups in two steps. The first step was formylation of both *ortho*-positions to give dialdehyde **11**. Subsequently, the aldehyde was reduced with NaBH_4_. Although the reduction afforded compound **12** in 92% yield in high purity without the need for chromatographic purification, the first formylation step proceeded in low yield, as compound **11** was obtained in only 35% yield.

The CH_2_ bisphenol **4** was prepared by selective de-*tert*-butylation from commercially available 6,6′-methylenebis-(2,4-di-*tert*-butylphenol) on a multigram scale (20 g) using a slightly modified original procedure [18]. The corresponding bis(hydroxymethyl) derivative **13** is available in good yield (~80%) from compound **4** by formylation ((CH_2_)_6_N_4_/TFA) [22] and subsequent reduction of the resulting dialdehyde with NaBH_4_.

Once we had the building blocks in hand, we tested both theoretically possible paths **A** (condensation **2** + **13**) and **B** (condensation **12** + **4**) leading to the desired macrocycle **7** (Scheme 3). The final macrocyclization step was performed under high dilution conditions using a dual syringe pump, allowing simultaneous addition of chloroform solutions of both building blocks to a large volume of chloroform containing PTSA as a catalyst. A four-hour addition followed by overnight reflux (argon atmosphere) led in both cases to a high yield of crude monothiacalix[4]arene **7**. Column chromatography on silica gel (eluent: CH_2_Cl_2_) afforded compound 7 in 66% yield for route **A** and 77% for route **B**. Both approaches thus provided macrocycle **7** in high yield, and both are therefore suitable for its synthesis. On the other hand, the higher yield of route **B** is disadvantaged by the necessity of preparing the starting bis(hydroxymethyl) derivative **12**, which consequently favors the condensation of compounds **2** + **13** (route A).

The ^1^H NMR spectrum of **7** (CDCl_3_, 400 MHz) showed four doublets in the aromatic part of the spectrum (7.05, 7.07, 7.20 and 7.46 ppm) with typical *meta*-coupling constants (*J* ≈ 2.5 Hz) corresponding to the signals of the aromatic CH bonds (Figures S5 and S6). The presence of two singlets of the *tert*-butyl groups around 1.21 ppm suggests a plane of symmetry for the resulting macrocycle possessing the *cone* conformation.

**Figure 1:**
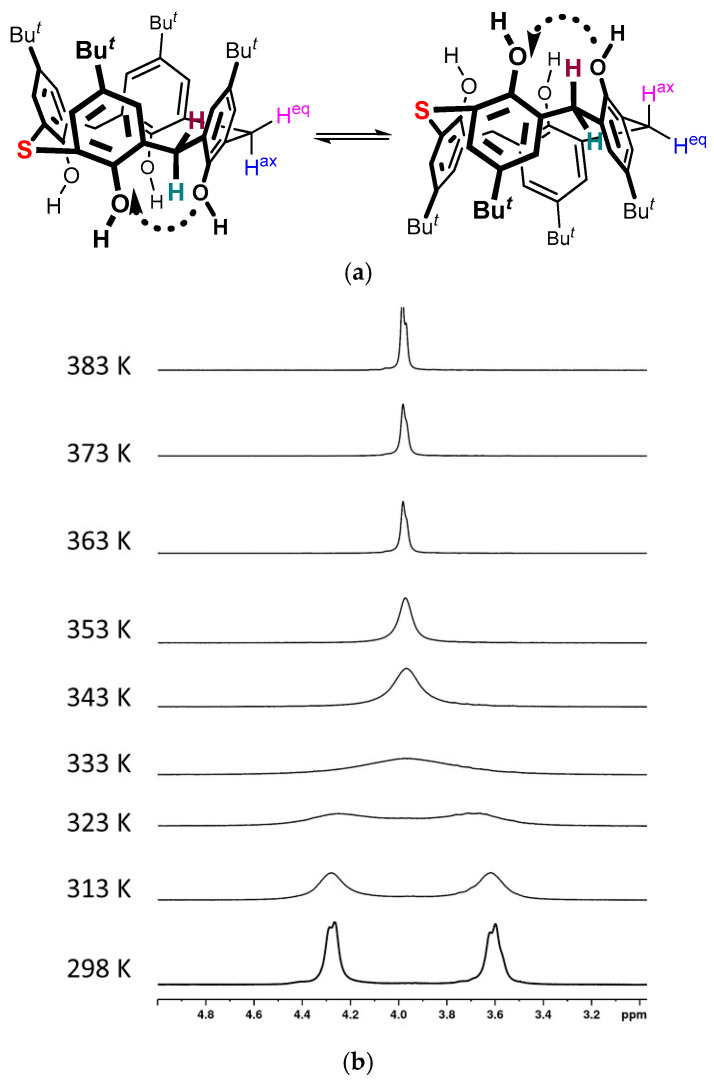
(**a**) The *cone–cone* interconversion of compound **7** with the chemical exchange between axial and equatorial protons. (**b**) The partial VT ^1^H NMR spectra of **7** (C_2_D_2_Cl_4_, 500 MHz) showing the area of CH_2_ bridges.

At room temperature, the very broad signals of the methylene bridges around 3.50 and 4.25 ppm indicate the presence of a dynamic process with kinetics comparable to the time scale of the NMR machine. This phenomenon can be attributed to the so-called *cone-inverted cone* interconversion, which is well known in calixarene chemistry [23,24]. The rapid inversion of the *cone* conformers of unsubstituted (with free OH groups) calix[4]arenes is manifested in ^1^H NMR spectra as the chemical exchange of the axial and equatorial protons (diastereotopic protons) of the CH_2_ bridging groups (Figure 1a).

Heating a solution of macrocycle **7** in 1,1,2,2-tetrachloroethane-*d*_2_ (Figure 1b) gradually led to broadening of both methylene bridge signals (2 × 2 broad overlapped doublets), and finally to the formation of two singlets corresponding to the situation under fast exchange conditions. The coalescence temperature (333 K) allowed the determination of the corresponding activation free energy Δ*G*^#^ of the inversion process (63 kJ·mol^−1^). This value is in good agreement with the barrier determined for the solution of **7** in CDCl_3_ (14.8 kcal·mol^−1^ ≈ 61.9 kJ·mol^−1^) [14].

**Figure 2:**
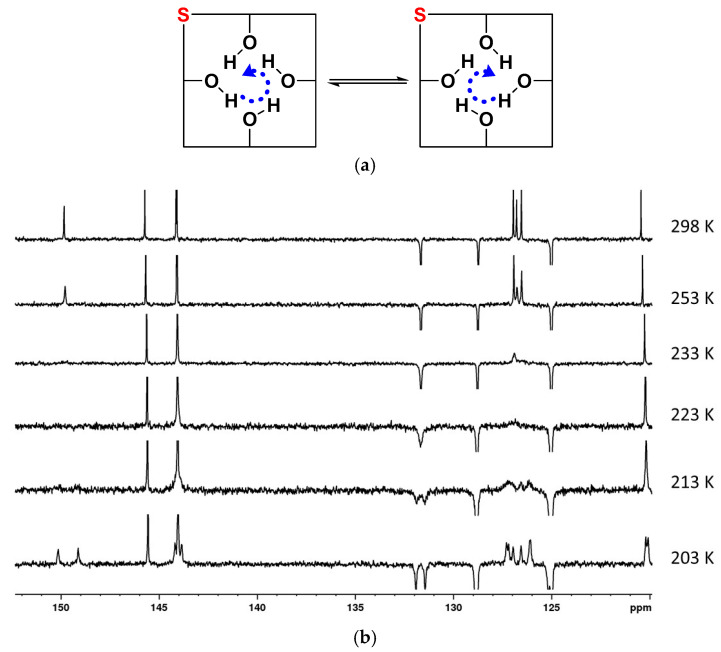
(**a**) *Flip-flop* motion of the hydrogen bond array. (**b**) Partial ^13^C APT NMR spectrum of **7** in the range of 203–298 K in CD_2_Cl_2_, 150 MHz (aromatic part of the spectrum).

Upon cooling the solution of compound **7** in CD_2_Cl_2_, the initially broad overlapped signals of OH protons (298 K) split into two broad singlets (253 K), which were then separated further into four broad singlets (two are merged together) (Figure S46). This phenomenon can be attributed to the so-called *flip-flop* motion [25,26] of the circular hydrogen bond array on the lower rim of the calixarene, in which the direction of the hydrogen bond changes (Figure 2a). This ultimately leads to non-equivalent signals of phenolic OH groups.

However, because the OH signals were too broad and it was not entirely clear which signals above and below the coalescence temperature correlate with each other, we used the ^13^C NMR spectrum of compound **7**, where a similar desymmetrization of the molecule occurs at quaternary carbons bearing OH groups. Thus, the signal at 150 ppm at 298 K split into two well-resolved signals (Δν = 131 Hz) at 203 K (Figure 2b). A similar splitting was observed also for other aromatic carbon resonances (see, e.g., the signal at around 132 ppm). The coalescence temperature (223 K) led to the ΔG^#^ value of 44 kJ·mol^−1^ for this *flip-flop* motion. It is noteworthy that this value is practically the same as that obtained [25] for the basic calix[4]arene **I** (43.9 kJ·mol^−1^). Thus, replacing one methylene bridge with a sulfur atom practically does not change the strength of the hydrogen bonds at the lower rim of macrocycle **7**.

**Scheme 4:**
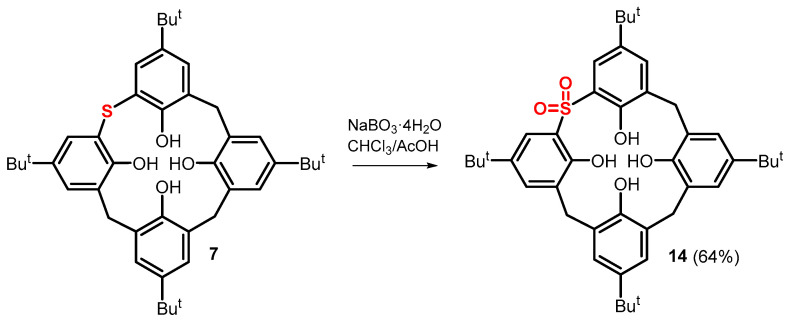
Oxidation of 2-thiacalix[4]arene.

To determine the effect of the oxidation state of sulfur on the dynamics of the molecule, we performed the oxidation of the sulfur bridge using an excess of sodium peroxoborate. The resulting sulfone **14** was obtained by preparative TLC in 64% yield (Scheme 4). The ^1^H NMR spectrum (500 MHz, CD_2_Cl_2_) again reflects the symmetry plane of the molecule with four doublets (7.60, 7.49, 7.17 and 7.150 ppm) in the aromatic region possessing a typical *meta* splitting pattern (*J* ≈ 2.5 Hz). Four very diffuse signals of phenolic hydroxyls (10.2 and 9.16 ppm) and the bridging CH_2_ (3.6 and 4.3 ppm) groups indicate dynamic behavior of the system at room temperature (Figures S34 and S35).

**Figure 3:**
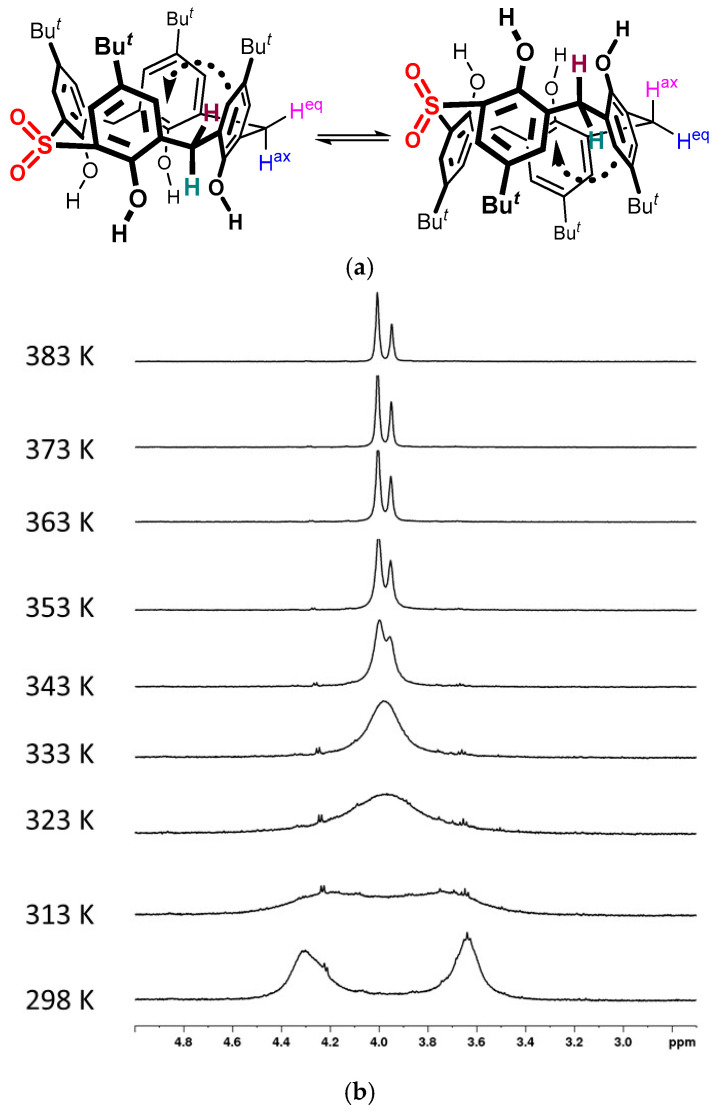
(**a**) The *cone–cone* interconversion of compound **14** with suggested chemical exchange between the CH_2_ protons. (**b**) The partial VT ^1^H NMR spectra of **14** (C_2_D_2_Cl_4_, 500 MHz) showing the area of CH_2_ bridges.

Heating of sulfone **14** in 1,1,2,2-tetrachloroethane-*d*_2_ (Figure 3) gradually led to broadening of methylene bridge signals, and finally to the formation of two singlets in a 2:1 ratio corresponding to the fast exchange conditions. The coalescence temperature (313 K) in this case corresponds to the free activation energy Δ*G*^#^ of the *cone–cone* interconversion of 60 kJ·mol^−1^, which is comparable to the barrier measured in chloroform [27]. The lower barrier (difference = 3 kJ·mol^−1^) of this equilibrium compared to that of the sulfur analogue **7** suggests that the cyclic hydrogen bond array is weaker due to the introduction of the sulfone group, leading to a more facile conformational change.

**Figure 4:**
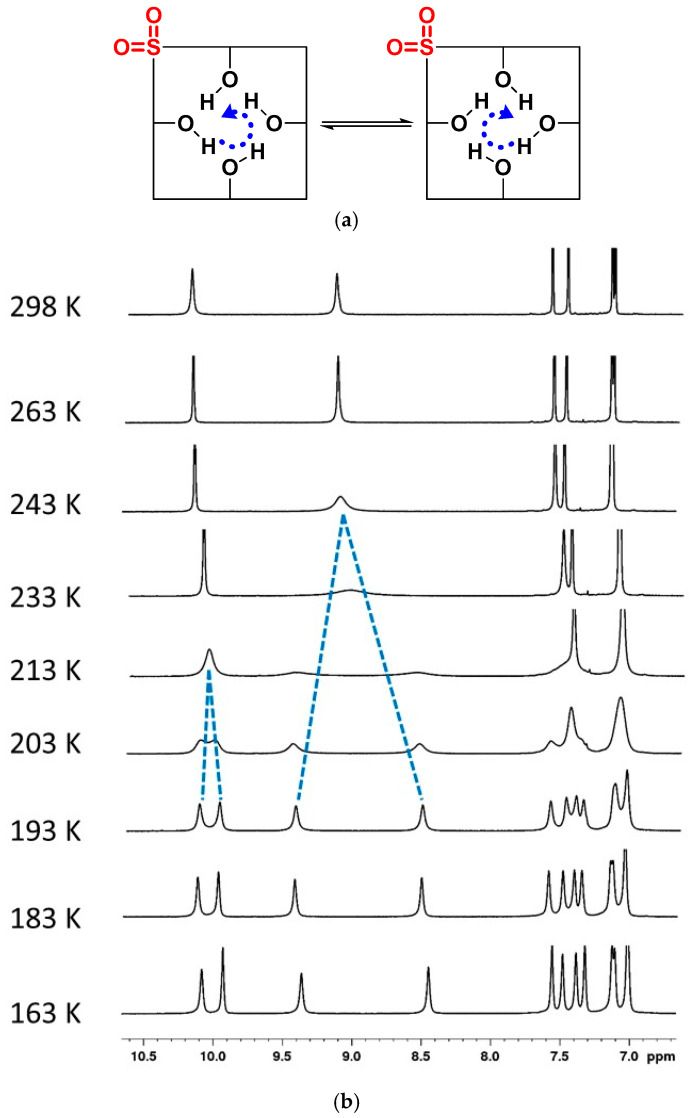
(**a**) *Flip-flop* motion of the hydrogen bond array. (**b**) Partial ^1^H NMR spectrum (CD_2_Cl_2_, 150 MHz) of **14** in the range of 163–298 K (aromatic part of the spectrum).

To confirm the negative effect of the SO_2_ group on the strength of hydrogen bonds at the lower rim of the calixarene, we performed low-temperature VT ^1^H NMR (500 MHz, CD_2_Cl_2_) measurements to determine the energy barrier for the *flip-flop* mechanism. As shown in Figure 4, both signals of phenolic OH groups (10.20 and 9.16 ppm) gradually transform into two new singlets, resulting from the asymmetric structure given by the directionality of hydrogen bonds. The corresponding coalescence temperatures (203 and 213 K, respectively) led to a Δ*G*^#^ of 40 kJ·mol^−1^ in both cases, which indeed indicated weaker hydrogen bonds of the macrocycle (compare the value of Δ*G*^#^ = 44 kJ·mol^−1^ for compound **7**).

**Figure 5:**
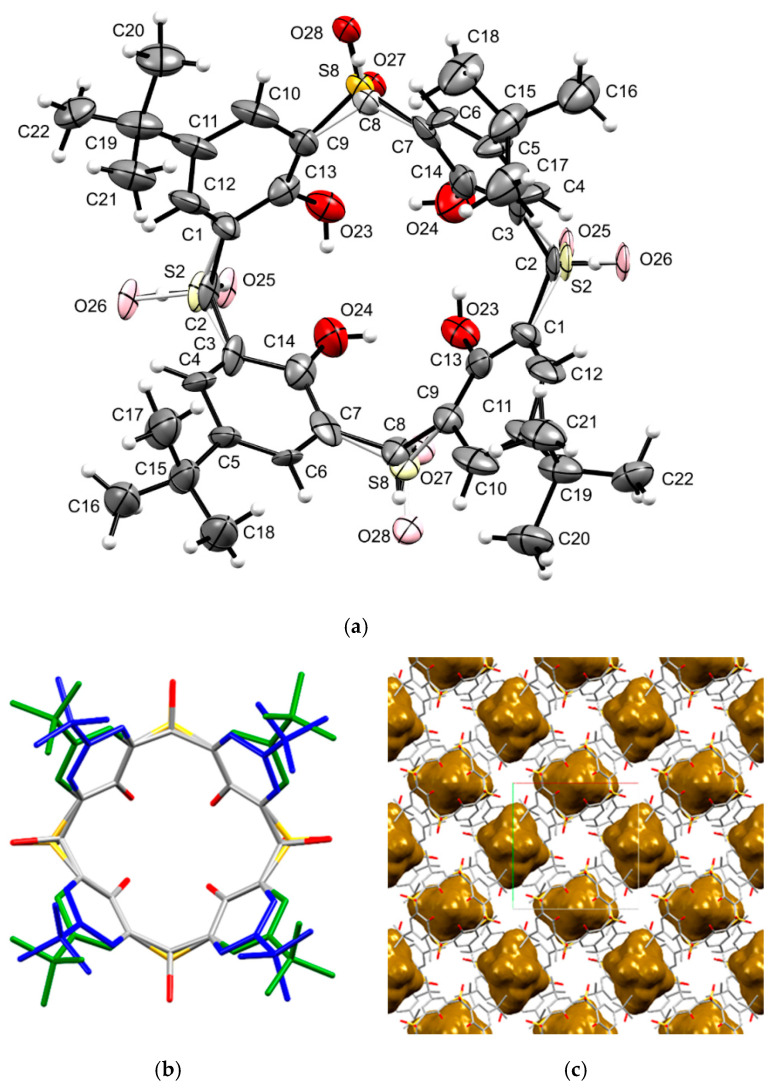
(**a**) ORTEP diagram of **14** at 50% probability with atom numbering. Disordered sulfonyl and methyl groups distinguished with lighter colour. Disorder of *tert*-butyl phenol groups omitted for clarity. (**b**) Disorder of flapping *tert*-butyl phenol groups (blue and green) in compound **14**. (**c**) Cavities of **14** occupied by cyclohexane displayed in a 3 × 3 cluster of the unit cells.

The final proof of the product structure was performed using single-crystal X-ray diffraction analysis of sulfone **14**. The macrocycle crystallized in a tetragonal crystal system, space group *P*-4, and adopted the *cone* conformation (Figure 5a). The sulfonyl bridge was statistically disordered in four positions with an occupancy of 25%. Four methylene group positions were occupied by 75%. Lowering the symmetry or twinning did not resolve the disorder problem, and no supercell reflections were observed. The symmetry of the macrocycle was too high to promote just one orientation of the molecule. The *tert*-butyl phenol groups reflected the changes in the structure with flapping between two positions by 7.8–15.4°, making the calix[4]arene more or less open (Figure 5b). The calixarene conformation variability was clearly triggered by the sulfonyl group, each surrounded by two flapped positions, altogether leading to each *tert*-butyl phenol having an occupancy of 0.5. The open cavity of the calixarene molecule was occupied by cyclohexane. The volume of the cavity was around 200 Å^3^, with two cavities covering 18% of the unit cell volume (calculated with respect to the disorder) (see Figure 5c). The OH hydrogen atoms were not found and were placed to follow the shortest donor–acceptor distance O23-H … O24, *d*(D-A) = 2.701(6) Å and O24-H … O23, *d*(D-A) = 2.717(6) Å. However, the sulfonyl group provided another hydrogen bond acceptor, although at random in 25% average due to the disorder: O23-H … O25, *d*(D-A) = 2.803(14) Å; O24-H…O27, *d*(D-A) = 2.943(14) Å. It was very likely that each molecule contained one bifurcated hydrogen bond, which ultimately resulted in a weakening of the cyclic hydrogen bond array, as suggested by the aforementioned VT NMR measurements.

## 3. Materials and Methods

### 3.1. General Experimental Procedures

All commercially obtained chemicals were purchased from commercial sources and used as received without further purification. Solvents were dried and distilled using conventional methods. NMR spectra were recorded on Agilent 400-MR DDR2 (Santa Clara, CA, USA), JEOL-ECZL400G (^1^H: 400 MHz, ^13^C: 100 MHz, Tokyo, Japan), Bruker Avance III 500 (^1^H: 500.13 MHz, ^13^C: 125.77 MHz, Bremen, Germany), and Bruker Avance III 600 (^1^H: 600.13 MHz, ^13^C: 150.92 MHz) spectrometers at 298 K. The deuterated solvents used are listed for each specific case. Chemical shifts (*δ*) are reported in parts per million (ppm) and were referenced to the residual peak of the solvent or TMS as an internal standard; coupling constants (*J*) are expressed in Hz. NMR data were processed and displayed using MestReNova (version 15.1.0) and TopSpin (version 3.6.5) software. The IR spectra were measured on an FT–IR spectrometer Nicolet iS50 (Thermo-Nicolet, Waltham, MA, USA) with a heatable Golden Gate Diamante ATR–Unit GladiATR (Specac, Orpington, UK). 64 Scans for one spectrum were co-added at a spectral resolution of 4 cm^–1^. Electrospray ionization mass spectra (ESI-MS) were recorded using a LTQ Orbitrap Velos—hybrid ion-trap-orbitrap (Thermo Scientific, Waltham, MA, USA). For melting point studies, Heiztisch Mikroskop—Polytherm A (Wagner & Munz, München, Germany) was used. The purity of the substances and the courses of the reactions were monitored by thin-layer chromatography (TLC) using silica gel 60 F_254_ on aluminum sheets (Merck, Lowe, NJ, USA) and analyzed at 254 nm. Column chromatography was carried out on silica gel 60 with a particle size of 0.063–0.200 mm (Merck, Darmstadt, Germany).

### 3.2. Synthetic Procedures

#### 3.2.1. Preparation of Compound **2**

##### From Compound **1**

*p*-*tert*-Butylphenol (100 g, 666.7 mmol) was dissolved in dry dichloromethane (600 mL) in a 1 L flask equipped with a magnetic stirrer and cooled in an ice bath. A solution of SCl_2_ (7.56 mL, 95.2 mmol, 80% in CH_2_Cl_2_) was added dropwise over 20 min at 0 °C. The reaction mixture was stirred for an additional 2.5 h at 0 °C, then neutralized with saturated NaHCO_3_ solution. The organic layer was dried over MgSO_4_, filtered, and concentrated under reduced pressure. The crude product was purified by repeated crystallization from cyclohexane (2–3×) followed by steam distillation to remove residual starting material (1–2 h). Extraction into CH_2_Cl_2_, drying, and evaporation afforded compound **2** as colorless crystals (21 g, 67%). ^1^H NMR (400 MHz, CDCl_3_, 298 K) δ 7.33 (d, *J* = 2.4 Hz, 2H, Ar-*H*), 7.24 (dd, *J* = 8.5, 2.4 Hz, 2H, Ar-*H*), 6.88 (d, *J* = 8.5 Hz, 2H, Ar-*H*), 6.02 (brs, 2H, -O*H*), 1.23 (s, 18H, *Bu^t^*). The data are consistent with the literature [9].

##### From Compound **10**

In 20 mL of dried acetone, 150 mg of sulfoxide **10** (0.43 mmol), 195 mg of NaI (1.30 mmol), and 455 mg of trifluoroacetic anhydride (2.16 mmol) were dissolved under an argon atmosphere. The reaction mixture was stirred for 20 min at room temperature and quenched by addition of saurated aqueous solutions of NaHCO_3_ and Na_2_SO_3_. The mixture was extracted with diethyl ether (3 × 15 mL), and the organic layer was dried over MgSO_4_. Evaporation of the solvent in vacuo gave 112 mg (78% yield) of pale yellowish solid **2**, which was identical to the product obtained above (Section From Compound **1**).

#### 3.2.2. Preparation of Compound **7**

##### Route A (from Compounds **2** and **13**)

*p*-Toluenesulfonic acid monohydrate (PTSA) (0.58 g, 3.05 mmol) was dissolved in 300 mL of dried chloroform in a 1 L round-bottom flask under an argon atmosphere. The solutions of substance **2** (0.50 g, 1.51 mmol) and substance **13** (0.56 g, 1.51 mmol) in 40 mL of dried CHCl_3_ were prepared in 50 mL syringes. Both solutions were then slowly added (4 h) to the flask under reflux using a programmable double syringe pump. After the addition, the reaction mixture was heated under reflux for another hour. After cooling, the crystallized PTSA was filtered off, the solvent was removed in vacuo, and the rest was purified by column chromatography on silica gel using dichloromethane as an eluent. The product was collected in the first fractions. For subsequent purification, the crude product was recrystallized from a mixture of chloroform and methanol to yield 0.67 g of pure compound 7 as white crystals (yield 66%). ^1^H NMR (500 MHz, CDCl_3_, 298 K) δ 10.13 (brs, 4H, -O*H*), 7.46 (d, *J* = 2.4 Hz, 2H, Ar-*H*), 7.21 (d, *J* = 2.4 Hz, 2H, Ar-*H*), 7.08 (d, *J* = 2.4 Hz, 2H, Ar-*H*), 7.06 (d, *J* = 2.4 Hz, 2H, Ar-*H*), 4.26 (brs, 3H, Ar-*CH_2_*-Ar), 3.52 (brs, 3H, Ar-*CH_2_*-Ar), 1.220 (s, 18H, *Bu^t^*), 1.216 (s, 18H, *Bu^t^*). ^13^C NMR (101 MHz, CDCl_3_, 298 K) δ 151.0, 146.7, 144.5, 144.4, 132.6, 129.6, 127.7, 127.6, 127.3, 126.1, 125.8, 121.3, 34.1, 34.0, 32.89, 32.6, 31.4, 31.3.

##### Route B (from Compounds **4** and **12**)

The reaction was carried out as in the previous case (route A), starting from 0.041 g of *p*-toluenesulfonic acid monohydrate (0.22 mmol) in 50 mL of dried chlorofom, 0.043 g of compound **12** (0.11 mmol), and 0.034 g of compound **4** (0.11 mmol) in 10 mL of CHCl_3_. The pure product **7** was obtained in 77% yield (0.056 g), identical with that from route A.

#### 3.2.3. Preparation of Compound **9**

The compound was prepared by modification of the original procedure [28]: Sulfur dichloride (0.52 g, 5 mmol) was added dropwise to a solution of 2,4-di-*tert*-butylphenol **8** (2.06 g, 10 mmol) in 20 mL of CH_2_Cl_2_ at 0 °C over 0.5 h. After addition, the cooling bath was removed, and the mixture was stirred for 3 h at room temperature. Water (20 mL) was carefully poured into the reaction mixture with stirring, and the organic layer was separated and washed twice with water. After drying over MgSO_4_, the solvent was removed in vacuo, and the residue was crystallized from MeCN to give 1.43 g of product **9** (64% yield) as a white crystalline solid. ^1^H NMR (400 MHz, CDCl_3_, 298 K) δ 7.26 (d, *J* = 2.4 Hz, 2H, Ar-*H*), 7.15 (d, *J* = 2.4 Hz, 2H, Ar-*H*), 6.48 (s, 2H, -O*H*), 1.41 (s, 18H, *Bu^t^*), 1.22 (s, 18H, *Bu^t^*). The data are consistent with the literature [17,28].

#### 3.2.4. Preparation of Compound **10**

*p-tert*-Butylphenol (1.50 g, 10 mmol) and 1.33 g of AlCl_3_ (10 mmol) were added to dry dichloromethane (20 mL), and the mixture was stirred for 30 min under ice cooling. The solution of thionyl chloride (0.59 g, 5 mmol) in 10 mL of dry DCM was added dropwise, the cooling bath was removed, and the mixture was stirred overnight (16 h). The solution was then poured into 50 mL of ice water. A semi-solid gummy substance was formed that adhered strongly to glass. Column chromatography on silica gel (DCM-AcOEt mixture) yielded 0.173 g of an oily product (10%), which solidified on prolonged standing, m.p. = 131–133 °C. ^1^H NMR (400 MHz, CDCl_3_, 298 K) δ 8.84 (brs, 2H, -O*H*), 7.37 (d, 2H, *J* = 8.6 Hz, 2H, Ar-*H*), 7.17 (s, 2H, Ar-*H*), 6.85 (d, 2H, *J* = 8.6 Hz, 2H, Ar-*H*), 1.23 (s, 18H, *Bu^t^*). ^13^C NMR (101 MHz, CDCl_3_, 298 K) *δ* 155.1, 143.5, 130.5, 123.0, 122.7, 118.4, 34.4, 31.4. HRMS (ESI^+^) (C_20_H_26_O_4_S) *m*/*z* calcd: 269.1495 [M + Na]^+^, found: 369.1500 [M + Na]^+^.

#### 3.2.5. Preparation of Compound **11**

Compound **2** (500 mg, 2 mmol) and hexamethylenetetramine (3.30 g, 24 mmol) were dissolved in trifluoroacetic acid (5 mL) and heated at 90 °C for 3 days. After this period, water (30 mL) was added, and the mixture was stirred at 80 °C for an additional 4 h. The cooled reaction mixture was extracted with dichloromethane, and the organic phase was dried over MgSO_4_. Purification by preparative TLC (DCM as eluent) afforded compound **11** as an orange solid (210 mg, 35%), m.p. = 101–104 °C. ^1^H NMR (400 MHz, CDCl_3_, 298 K) δ 11.28 (s, 2H), 9.90 (s, 2H), 7.54 (s, 2H), 7.48 (s, 2H), 1.24 (s, 18H). ^13^C NMR (101 MHz, CDCl_3_, 298 K) *δ* 196.5, 158.3, 143.4, 137.8, 129.7, 121.6, 120.2, 34.3, 31.2. IR (ATR) *ν* (cm^−1^) 2957, 2925, 2862, 2650. HRMS (ESI^+^) (C_22_H_26_O_4_S) *m*/*z* calcd: 409.14440 [M + Na]^+^, found: 409.14491 [M + Na]^+^.

#### 3.2.6. Preparation of Compound **12**

##### From Compound **2**

Compound **12** was prepared in 70% yield using the published procedure [9] starting from 5 g of bisphenol **2** (direct hydroxymethylation with formaldehyde and KOH).

##### From Compound **11**

Compound **11** (0.68 g, 1.84 mmol) was dissolved in 10 mL of tetrahydrofuran and NaBH_4_ (0.28 g, 7.4 mmol) was gradually added under constant stirring and ice cooling. The mixture was then stirred at room temperature for 2 h. The reaction mixture was quenched with 5 mL of water, and the pH was adjusted to neutral by adding 1 molar HCl. The product was extracted with dichloromethane (3 × 15 mL). After drying with MgSO_4_ and evaporation, the pure product **12** (0.63 g, 92% yield) was obtained as a white powder. ^1^H NMR (400 MHz, CDCl_3_, 298 K) δ 10.01 (brs, 2H, O*H*), 7.34 (d, *J* = 2.4 Hz, 2H, Ar-*H*), 7.09 (d, *J* = 2.4 Hz, 2H, Ar-*H*), 4.74 (s, 4H, Ar-C*H_2_*-OH), 2.80 (brs, 2H, CH_2_-O*H*), 1.23 (s, 18H, *Bu^t^*). The data are consistent with the literature [9].

#### 3.2.7. Preparation of Compound **14**

Compound 7 (200 mg, 0.29 mmol) was treated with ten equivalents of NaBO_3_·4H_2_O (447 mg, 2.9 mmol) in a mixture of chloroform (8 mL) and acetic acid (10 mL). The mixture was stirred overnight at 50 °C. The reaction was neutralized with NaHCO_3_ and evaporated to dryness using a vacuum evaporator, the solid was dissolved in DCM and dried over MgSO4. The solvent was removed, and the solid was dissolved in a cyclohexane:ethyl acetate 1:1 (v:v) mixture and was left to evaporate slowly. After a few days, clear crystals were collected to give compound **14** in 64% yield, m.p. > 300 °C. ^1^H NMR (CD_2_Cl_2_, 600.1 MHz, 298 K) *δ* 10.2 (brs, 2H, O*H*), 10.16 (brs, 2H, O*H*), 7.60 (d, 2H, *J* = 2.5 Hz, Ar-*H*), 7.49 (d, 2H, *J* = 2.5 Hz, Ar-*H*), 7.17 (d, 2H, *J* = 2.4 Hz, Ar-*H*), 7.15 (d, 2H, *J* = 2.4 Hz, Ar-*H*), 4.20–4.40 (brs, 3H, Ar-*CH_2_*-Ar), 3.60 (brs, 3H, Ar-*CH_2_*-Ar), 1.253 (s, 18H, *Bu^t^*), 1.245 (s, 18H, *Bu^t^*) ppm. ^13^C NMR (CD_2_Cl_2_, 150.9 MHz, 298 K) *δ* 150.2, 146.4, 145.2, 144.2, 133.8, 129.2, 127.6, 126.3, 126.2, 126.0, 125.7, 124.7, 34.2, 33.9, 32.0, 31.2, 31.1, 30.8 ppm. IR (ATR) *ν* (cm^−1^) 3265, 2957, 2869, 1484. HRMS (ESI^+^) (C_43_H_54_O_6_S) *m*/*z* calcd: 721.3533 [M + Na]^+^, found: 721.3533 [M + Na]^+^.

### 3.3. X-Ray Measurements

#### Crystallographic Data for **14**

C_43_H_54_O_6_S·C_6_H_12_, M = 783.12 g·mol^−1^, tetragonal system, space group *P-4*, *a* = 12.7258(3) Å, *c* = 13.6553(5) Å, *Z* = 2, *V* = 2211.42(13) Å^3^, *D_c_* = 1.176 g·cm^−3^, *μ*(Cu-Kα) = 1.017 mm^−1^, crystal dimensions of 0.08 × 0.19 × 0.23 mm. Data were collected at 180 (2) K on a Bruker D8 Venture Photon II 7 diffractometer with Incoatec microfocus sealed tube Cu-Kα radiation. The data were integrated, scaled and corrected for absorption using Apex4. [29] The structure was solved by SIR92 [30] and anisotropically refined by full matrix least squares on *F* squared using CRYSTALS [31] to a final value of *R*_1_ 0.106 (3716 reflections) and *wR* = 0.261 (3930 independent reflections), *θ_max_* = 69.7°, 417 refined parameters, and 365 restraints. The hydrogen atoms bonded to carbon atoms were placed in calculated positions and refined with riding constraints. The hydrogen atoms bonded to oxygen atoms were not found; thus, they were placed to follow the shortest hydrogen bond 0.82 Å from the pivot oxygen atoms, and further refinement was unstable. The disordered functional group positions were found in difference electron density maps and refined with restrained geometry. Eventual minor deformations of the low-rim calix skeleton were neglected. DELU and SIMU restraints were used to keep overlapping ADP’s acceptable. MCE [32] was used for visualization of electron density maps. The occupancy of disordered functional groups was initially refined constrained to full; during final stages, it was refined fixed at 0.25 (sulfonyl), 0.5 (*tert*-butyl phenol) and 0.75 (methylene). It was extremely difficult to obtain single-crystal suitable for X-ray data collection from experiments usually resulting in subtle polycrystalline aggregates. The data collection of the best single-crystal, obtained from repeated crystal growth attempts, took five days. The structure was deposited in the Cambridge Structural Database under number CCDC 2469735.

## 4. Conclusions

The article describes a simple and scalable preparation of 2-monothiacalix[4]arene **7**, the simplest representative of the mixed-bridged (CH_2_ and S) calix[4]arenes. The synthesis is based on the condensation of linear building blocks (bisphenols), which are relatively readily available, and allows, depending on the circumstances, the use of two alternative reaction routes that provide product **7** in high yield. The dynamic behaviour of the macrocyclic skeleton was investigated using NMR spectroscopy at variable temperatures. High-temperature measurements showed that compound **7** undergoes a *cone*–*cone* equilibrium with activation free energy Δ*G*^#^ of the inversion process of 63 kJ·mol^−1^. Interestingly, the same barrier for the oxidized sulfone derivative **14** shows a value of 60 kJ·mol^−1^, indicating weakened hydrogen bonds at the lower rim of the calixarene. The same was also confirmed at low temperatures, when barriers to changing the direction of the cyclic hydrogen bond arrays (*flip-flop* mechanism) were determined (compare ΔG^#^ = 44 kJ·mol^−1^ for **7** vs. ΔG^#^ = 40 kJ·mol^−1^ for **14**).

## Data Availability

All experimental data are provided in the Supplementary Information.

## Supplementary Materials

The following supporting information can be downloaded at: https://www.mdpi.com/article/10.3390/molecules30153145/s1, Spectral characterization of all new compounds (^1^H NMR, ^13^C NMR, HRMS, IR), VT NMR experiments, X-ray structure.
