# Supplementary material for: Synthesis of Monothiacalix[4]arene Using the Fragment Condensation Approach
Source: Molecules. 2025 Jul 27;30(15):3145. doi: 10.3390/molecules30153145 (PMC12348300; doi:10.3390/molecules30153145)
Supplement: Supplementary file 1 [file molecules-30-03145-s001.zip › molecules-3768854-supplementary.pdf]

Supporting Information

Synthesis od Monothiacalix[4]arene Using the Fragment Condensation Approach

Daniel Kortus [1], Oliver Moravec [1], Michal Churý [1], Kamil Mamleev [1], Jan Čejka [2], Hana Dvořáková [3] and Pavel Lhoták [1,*]

[1] Department of Organic Chemistry, University of Chemistry and Technology, Prague (UCTP), Technická 5, 166 28 Prague 6, Czech Republic.
[2] Department of Solid State Chemistry, UCTP, Technická 5, 166 28 Prague 6, Czech Republic.
[3] Laboratory of Nuclear Magnetic Resonance Spectroscopy, UCTP, Technická 5, 166 28 Prague 6, Czech Republic.

* Correspondence: lhotakp@vscht.cz; Tel.: +420-440225055

Table of Contents

1. Spectral characterization of compounds 2

2. Crystallographic data 24

3. Variable Temperature NMR 26

1. Spectral characterization of compounds

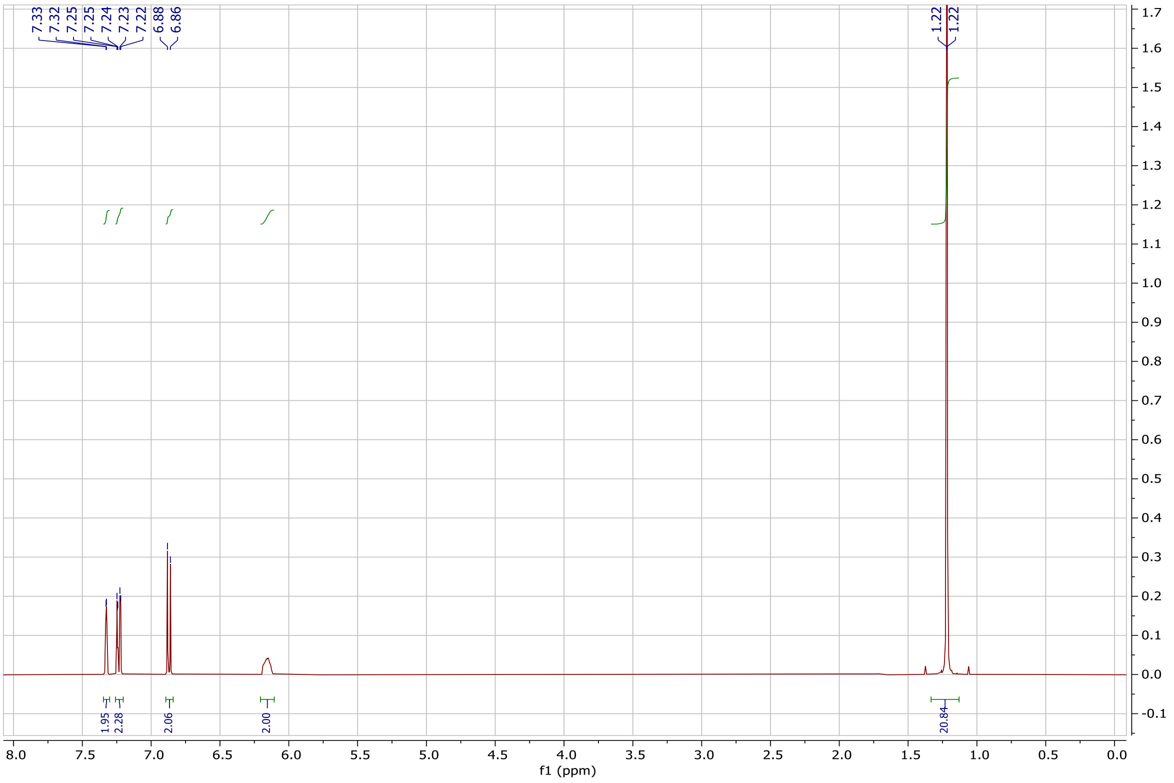

Figure S1: ^{1}H NMR of compound **2** ($CDCl_3$, 400 MHz, 298 K)

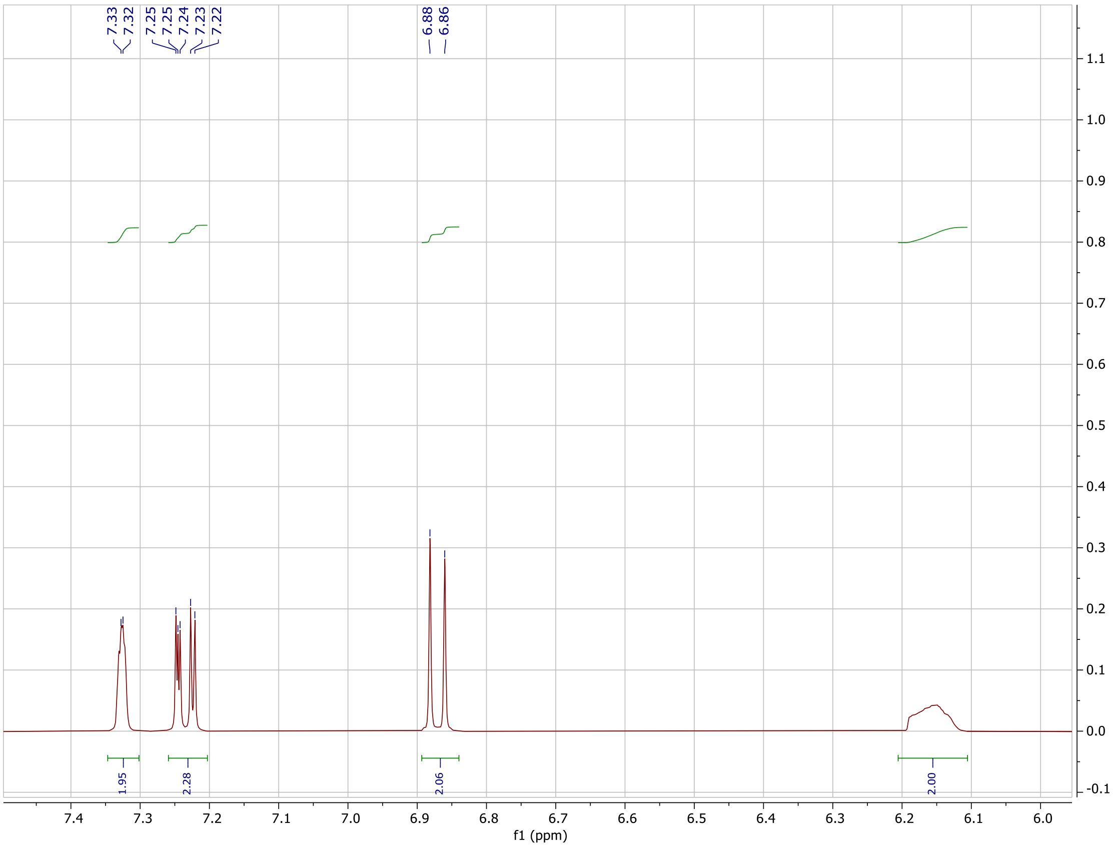

Figure S2: ^{1}H NMR of compound **2** – aromatic region ($CDCl_3$, 400 MHz, 298 K)

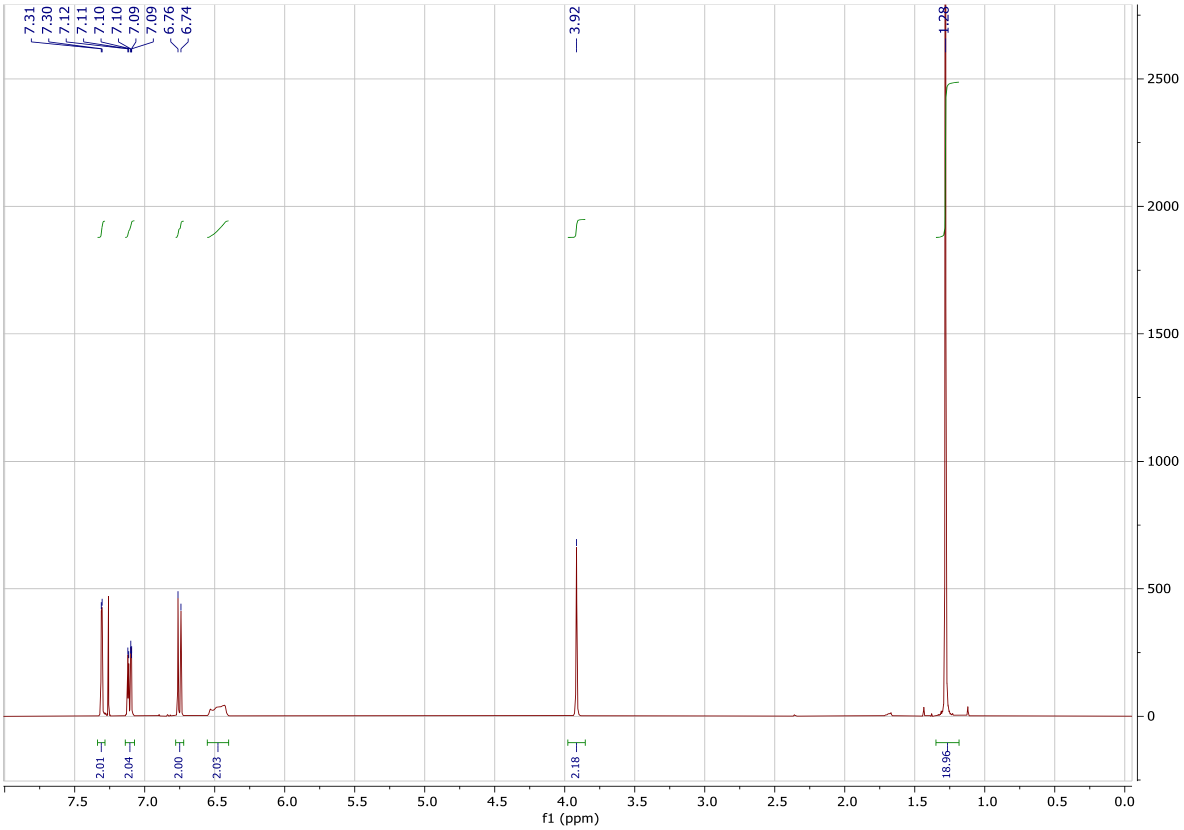

Figure S3: ^{1}H NMR of compound **4** ($CDCl_3$, 400 MHz, 298 K)

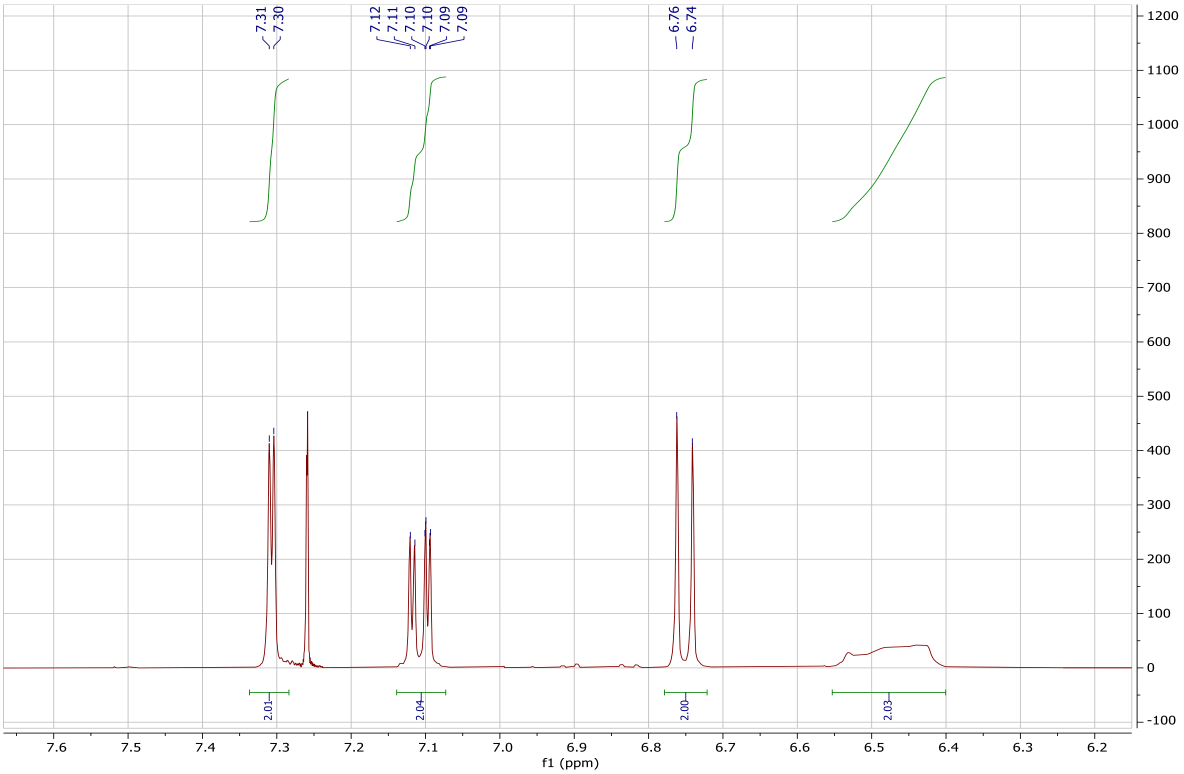

Figure S4: ^{1}H NMR of compound **4** – aromatic region ($CDCl_3$, 400 MHz, 298 K)

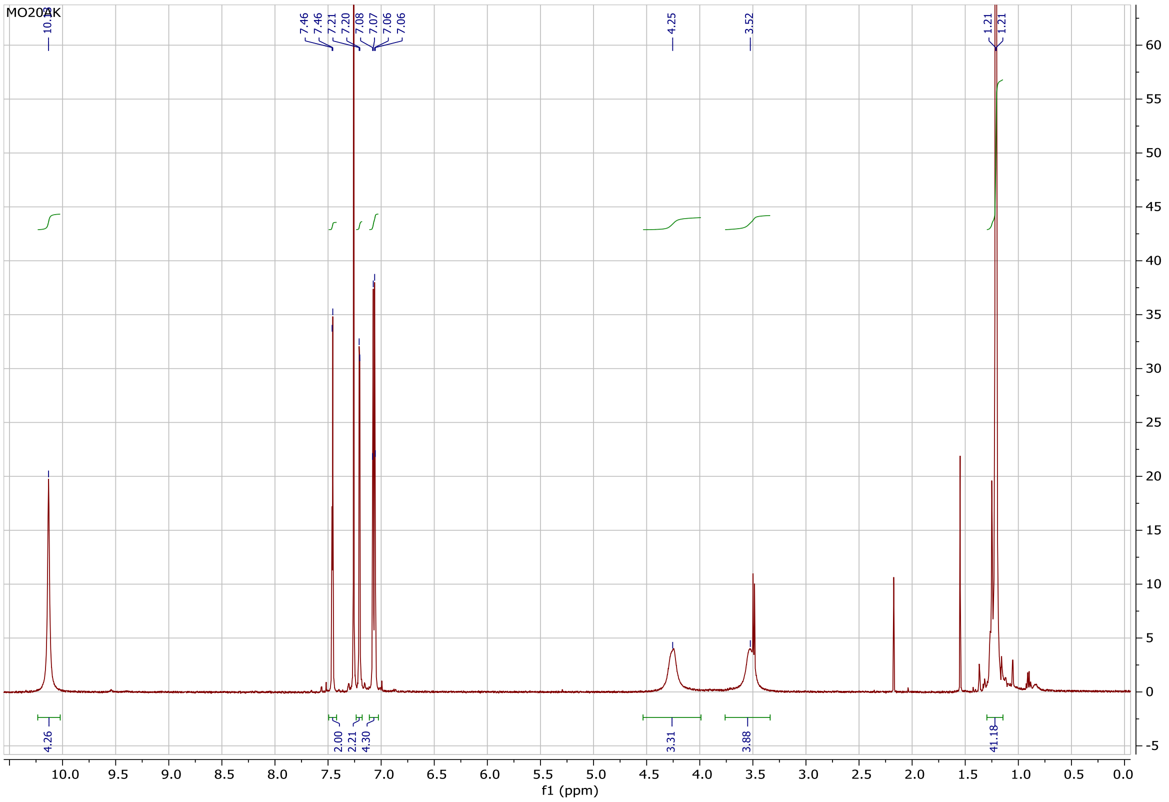

Figure S5: ^{1}H NMR of compound **7** ($CDCl_3$, 400 MHz, 298 K)

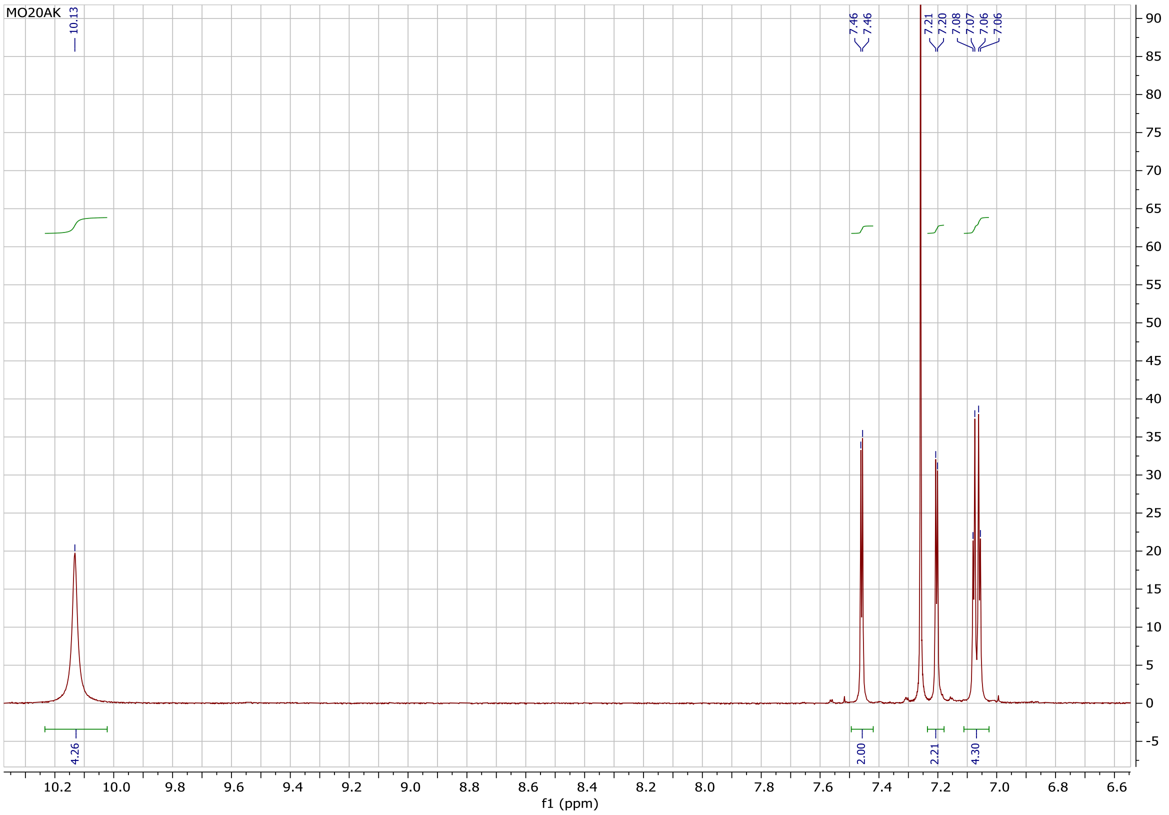

Figure S6: ^{1}H NMR of compound **7** – aromatic region ($CDCl_3$, 400 MHz, 298 K)

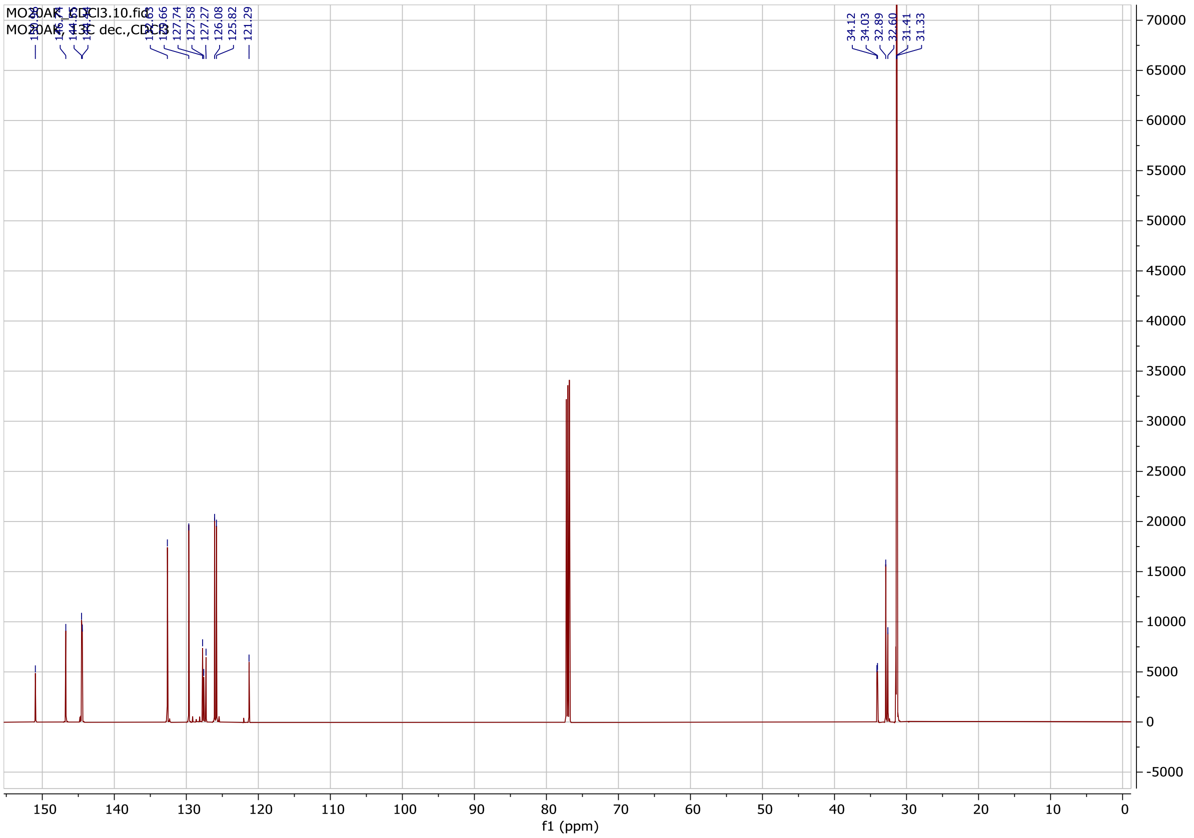

Figure S7: ^{13}C NMR of compound **7** ($CDCl_3$, 100 MHz, 298 K)

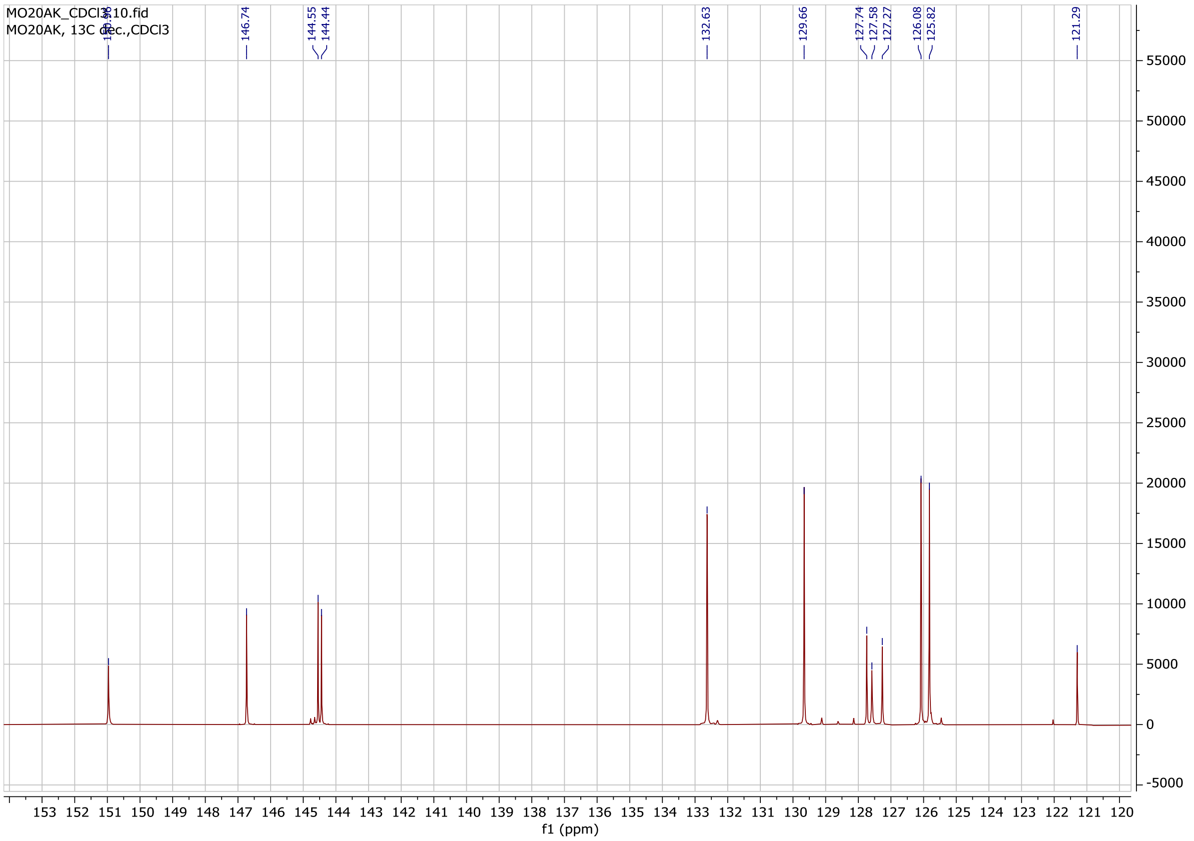

Figure S8: ^{13}C NMR of compound **7** – aromatic region ($CDCl_3$, 100 MHz, 298 K)

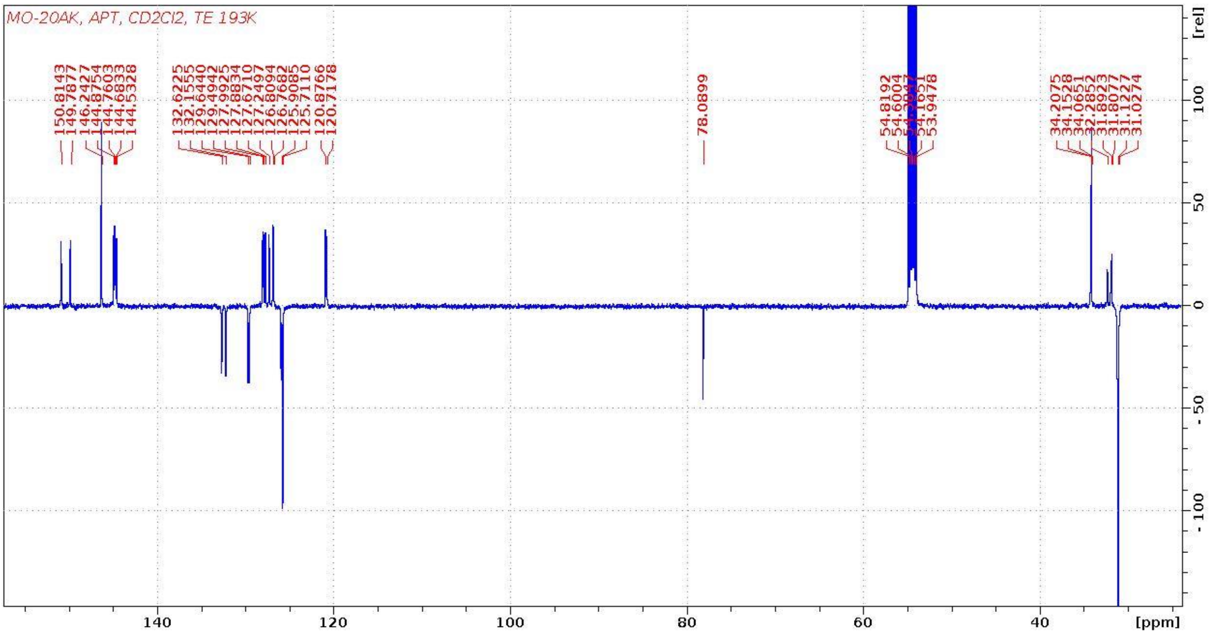

Figure S9: ^{13}C APT NMR of compound **7** – full spectrum (CD_2Cl_2, 150 MHz, 193 K)

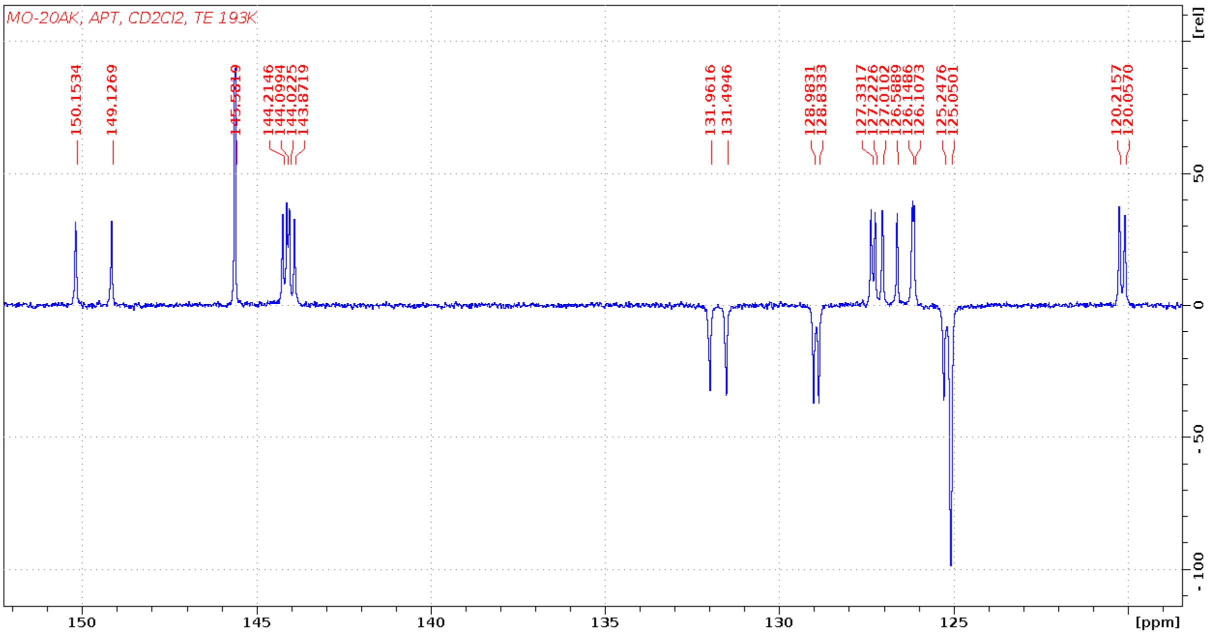

Figure S10: ^{13}C APT NMR of compound **7** – aromatic region (CD_2Cl_2, 150 MHz, 193 K)

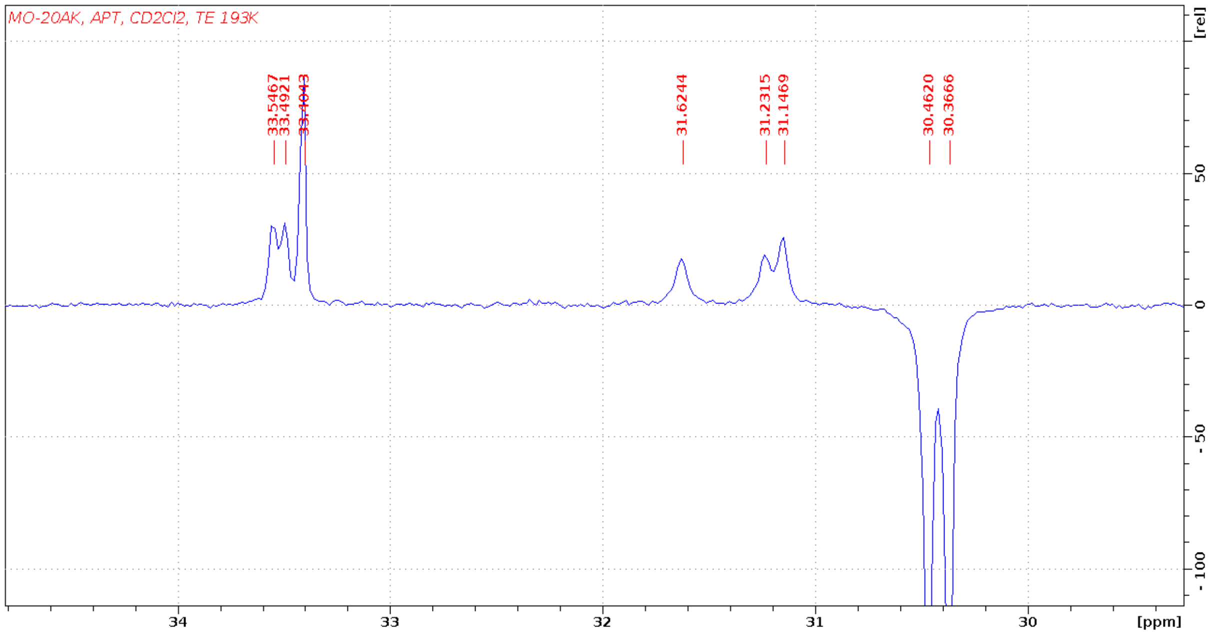

Figure S11: ^{13}C APT NMR of compound **7** – aliphatic region (CD_2Cl_2, 150 MHz, 193 K)

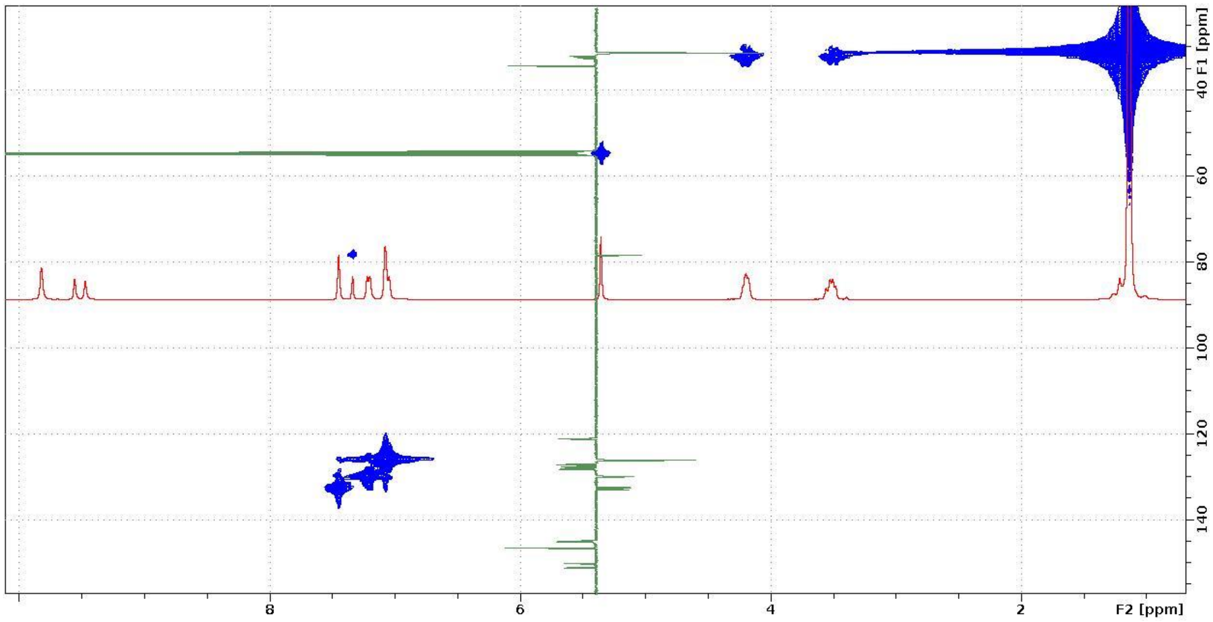

Figure S12: HMQC NMR of compound **7** – full spectrum (CD_2Cl_2, 193 K)

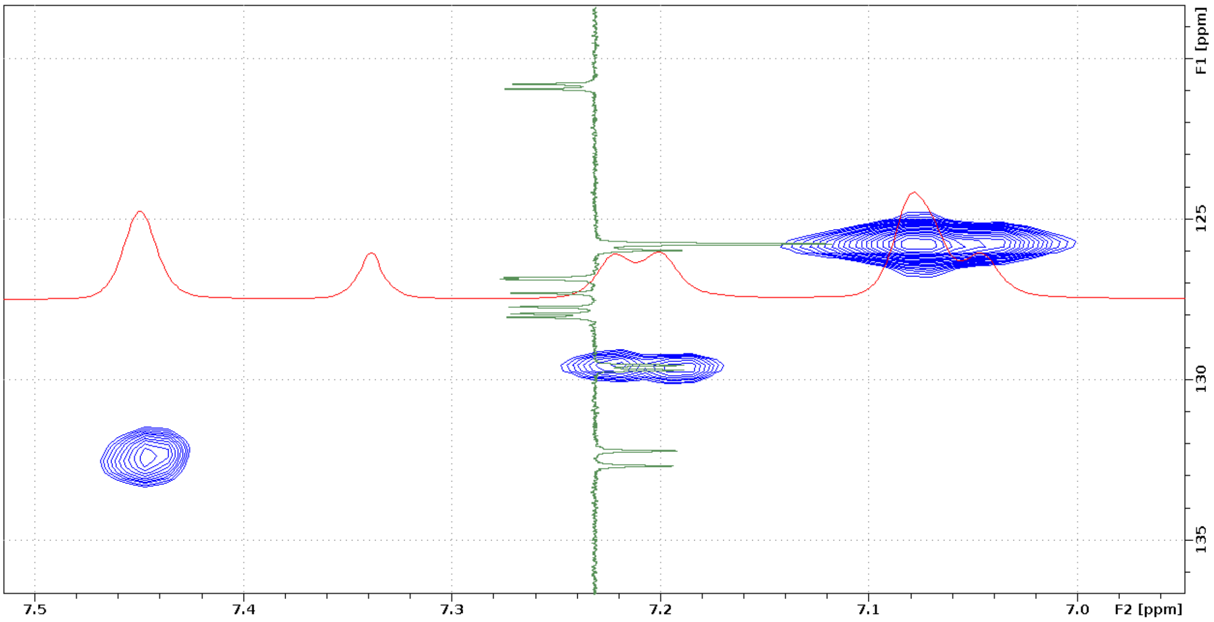

Figure S13: HMQC NMR of compound **7** – aromatic region (CD_2Cl_2, 193 K)

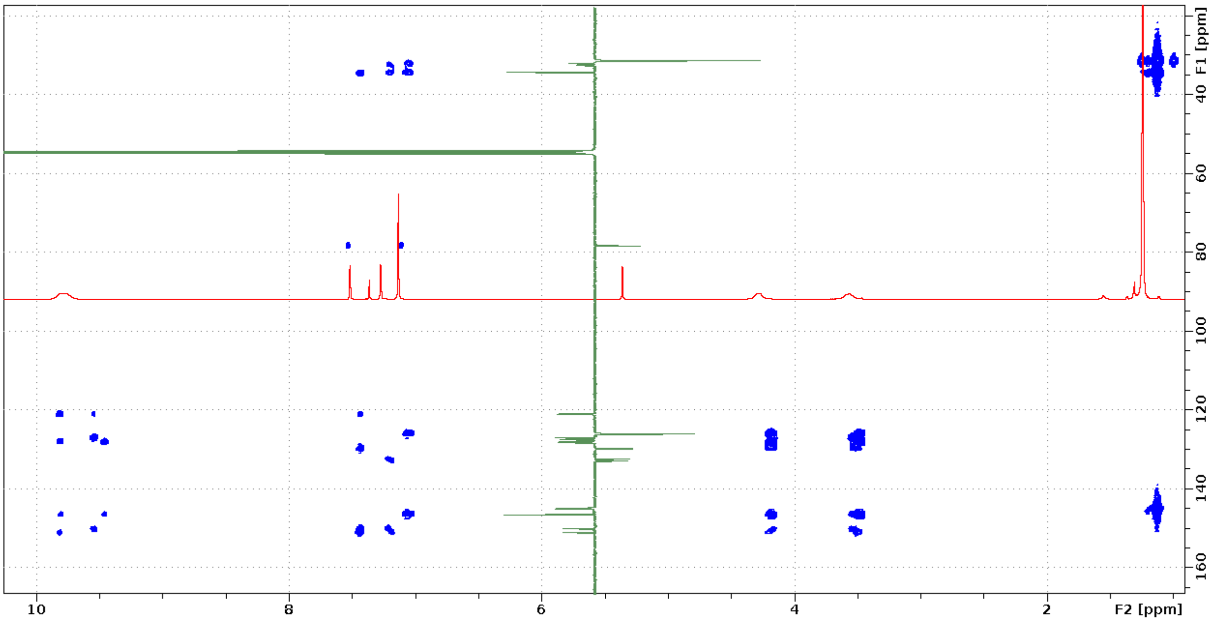

Figure S14: HMBC NMR of compound **7** – full spectrum (CD_2Cl_2, 193 K)

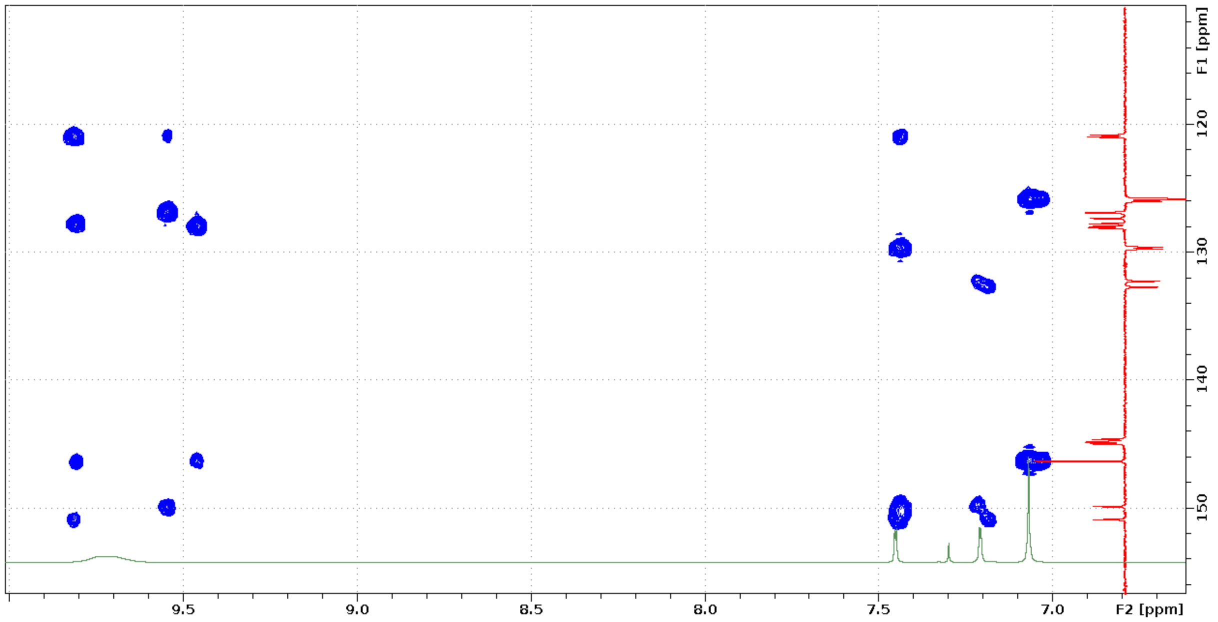

Figure S15: HMBC NMR of compound **7** – aromatic region (CD_2Cl_2, 193 K)

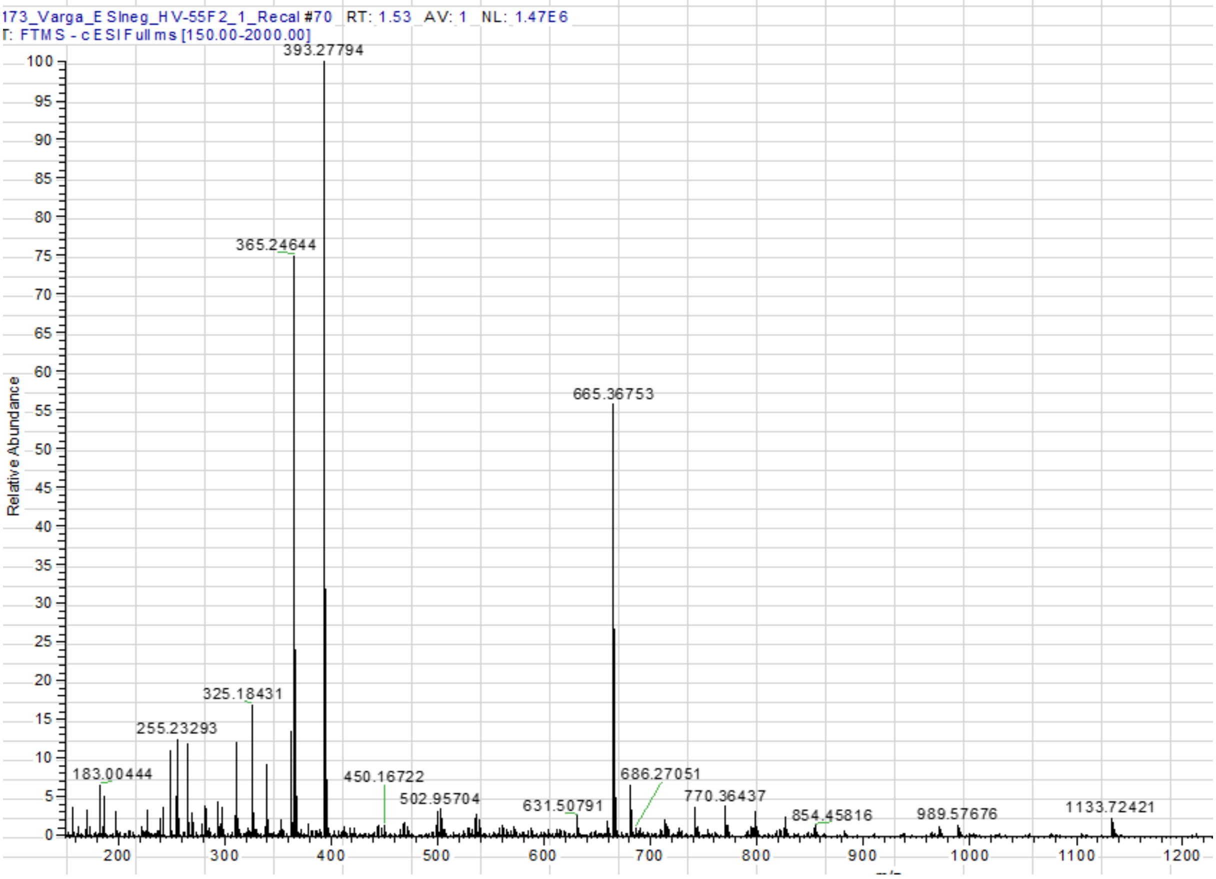

Figure S16: HRMS of compound **7** (ESI$^-$)

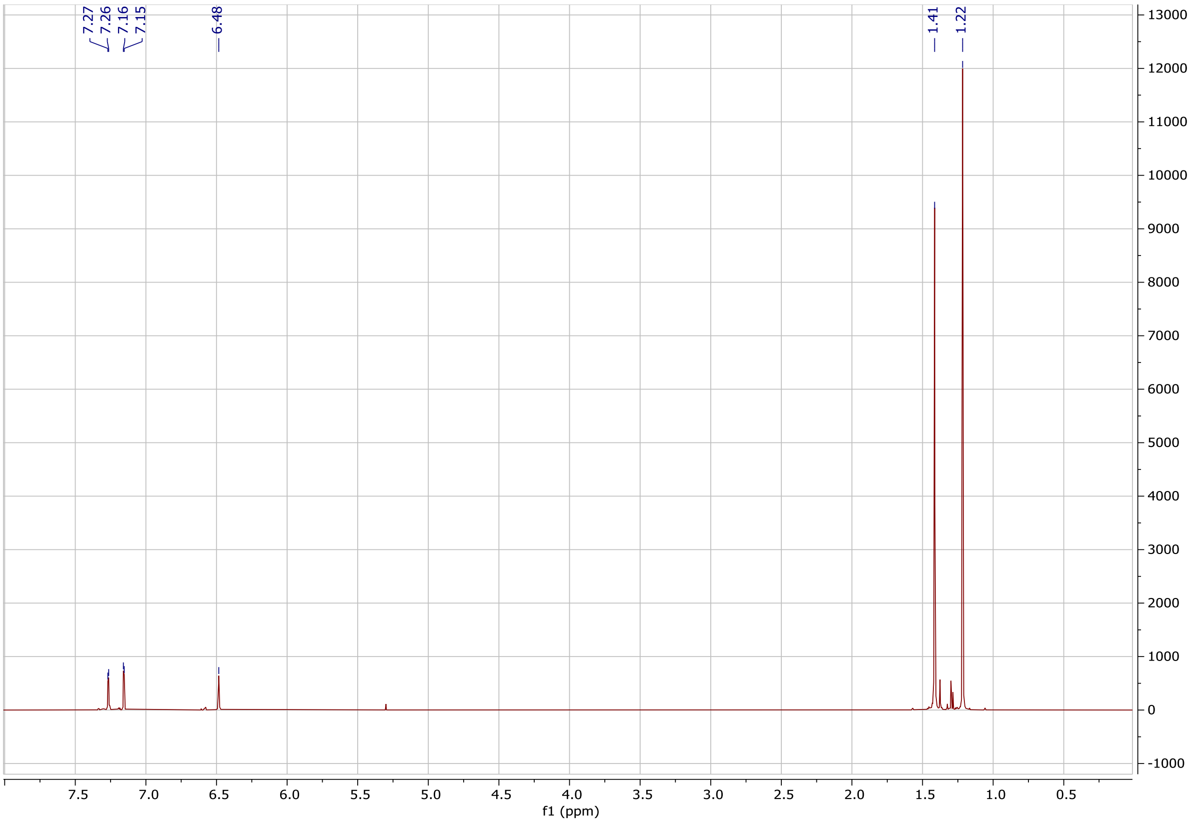

Figure S17: ^{1}H NMR of compound **9** ($CDCl_3$, 400 MHz, 298 K)

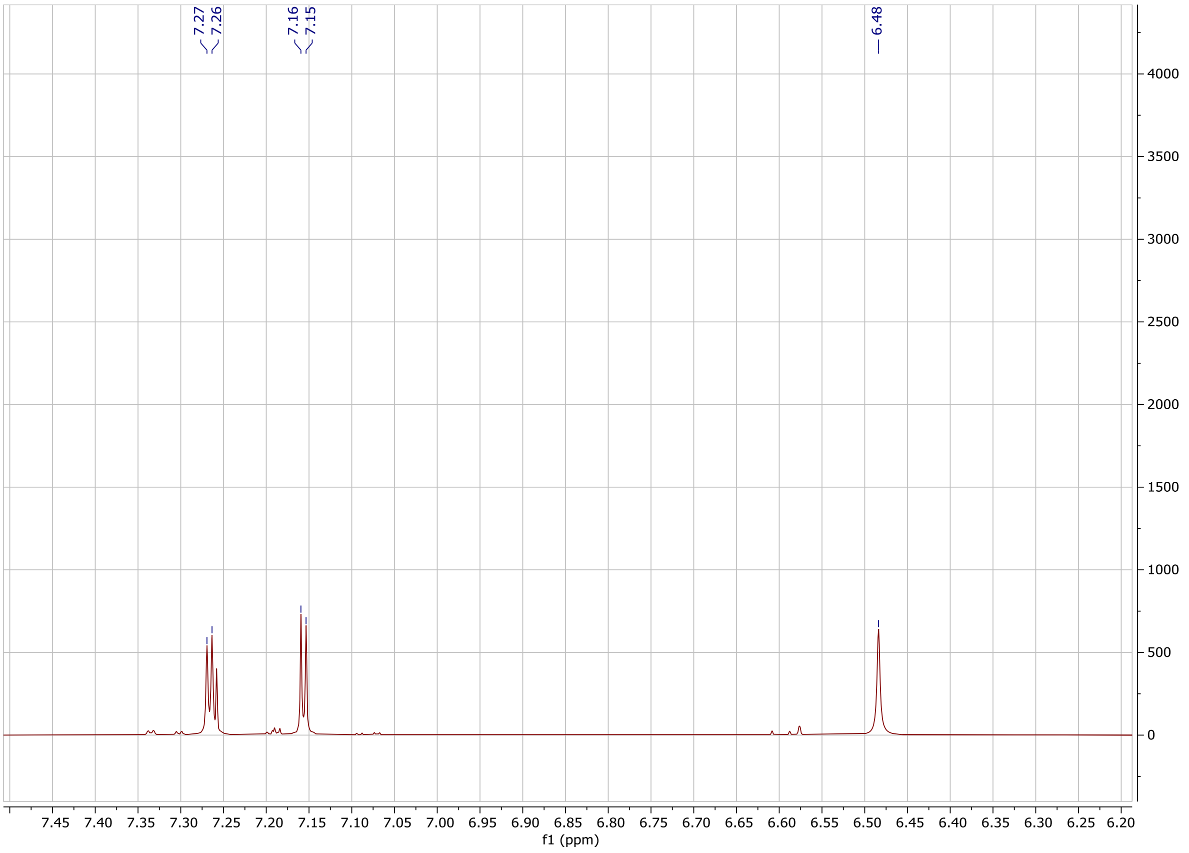

Figure S18: ^{1}H NMR of compound **9** – aromatic region ($CDCl_3$, 400 MHz, 298 K)

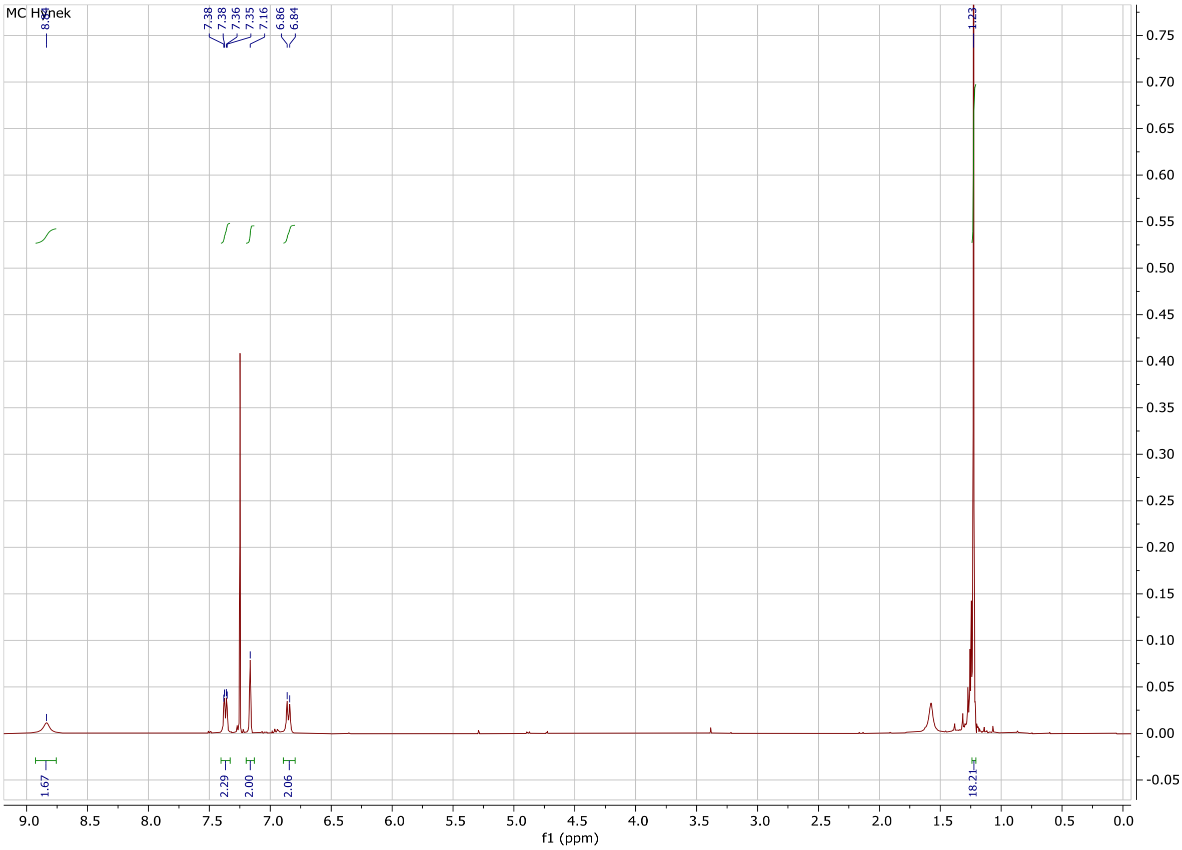

Figure S19: ^{1}H NMR of compound **10** – full spectrum ($CDCl_3$, 400 MHz, 298 K)

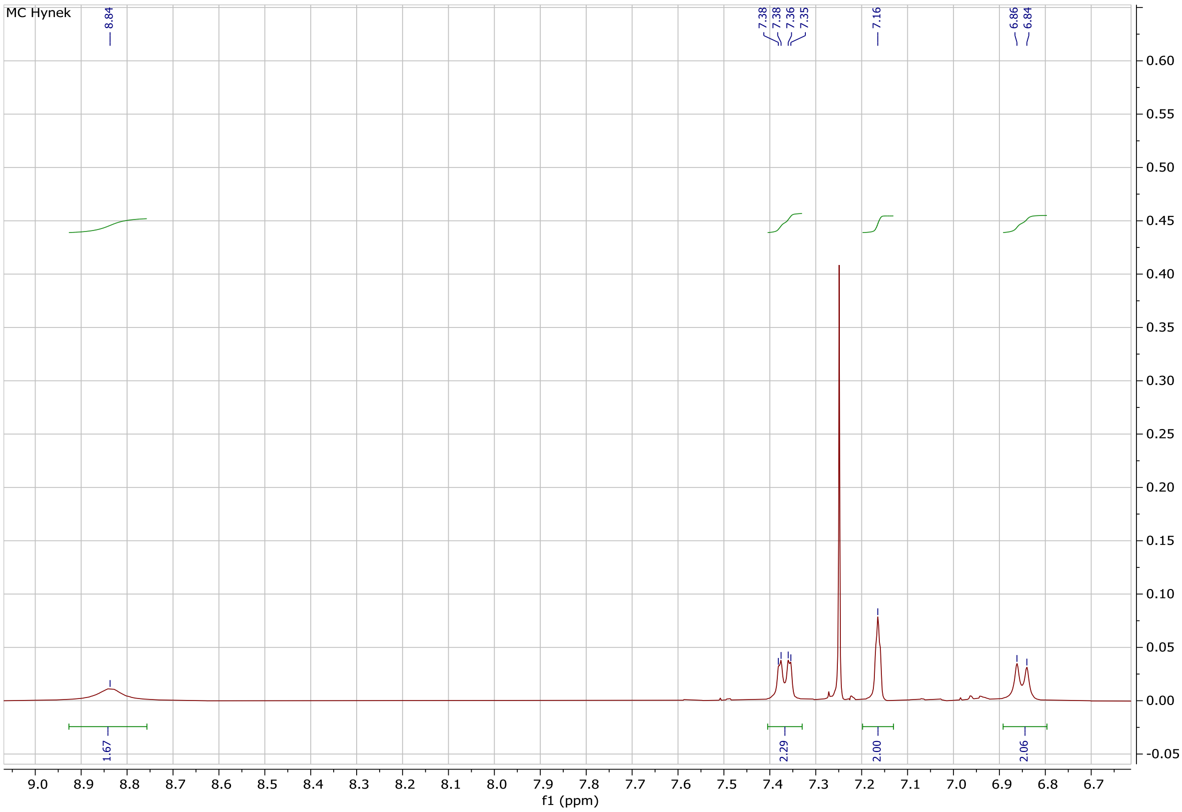

Figure S20: ^{1}H NMR of compound **10** – aromatic region ($CDCl_3$, 400 MHz, 298 K)

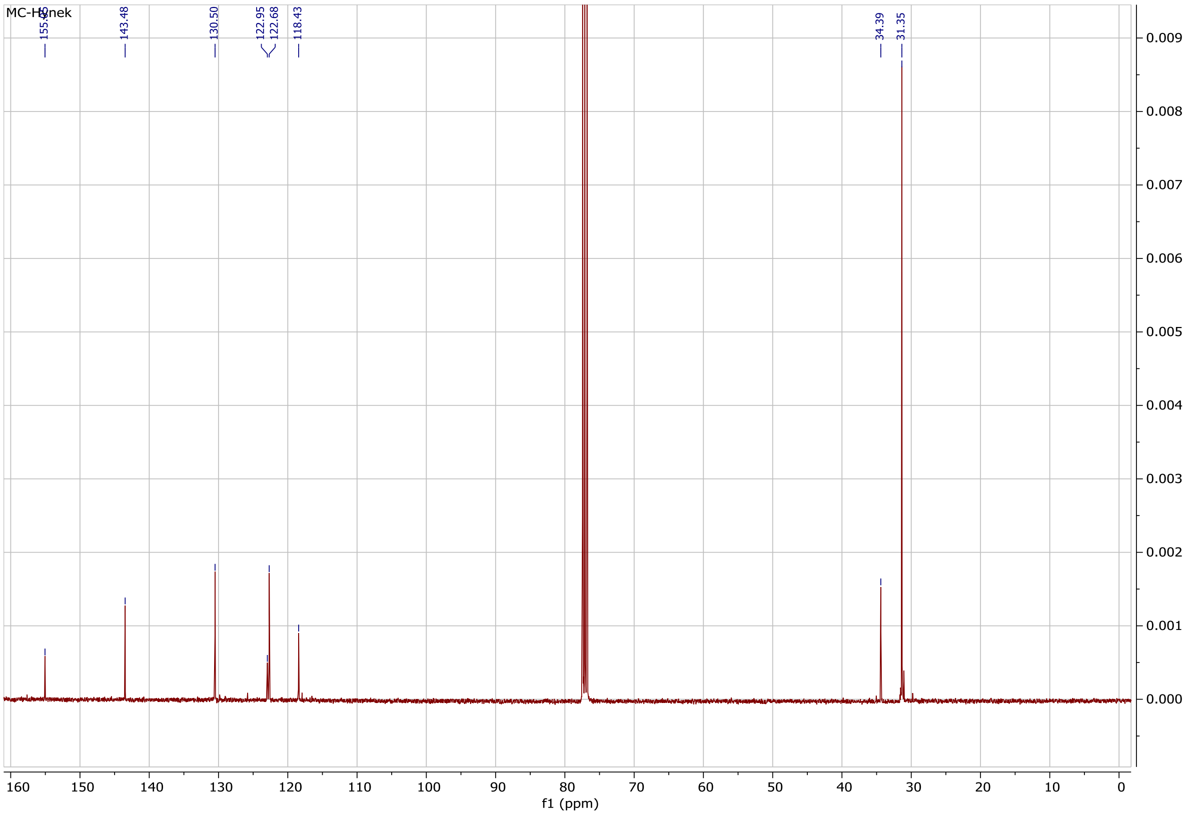

Figure S21: ^{13}C NMR of compound **10** – full spectrum ($CDCl_3$, 100 MHz, 298 K)

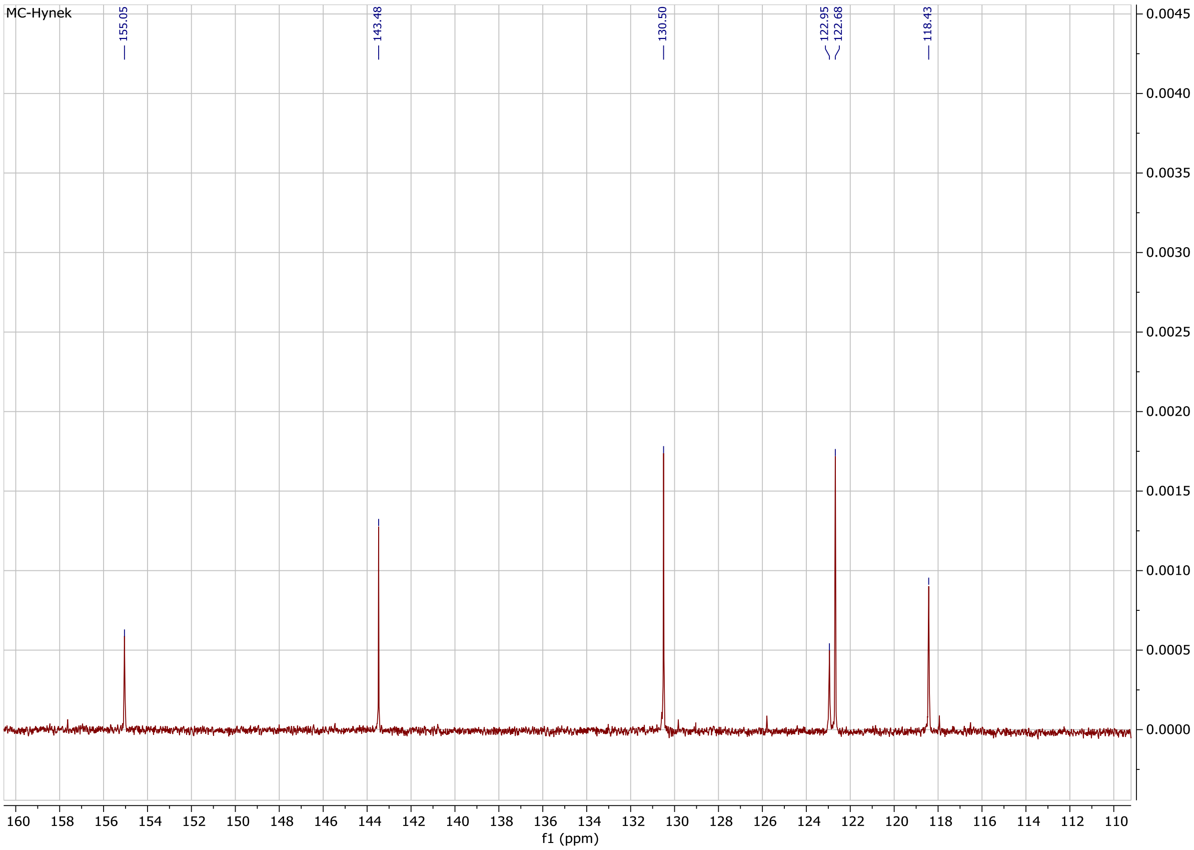

Figure S22: ^{13}C NMR of compound **10** – aromatic reagion ($CDCl_3$, 100 MHz, 298 K)

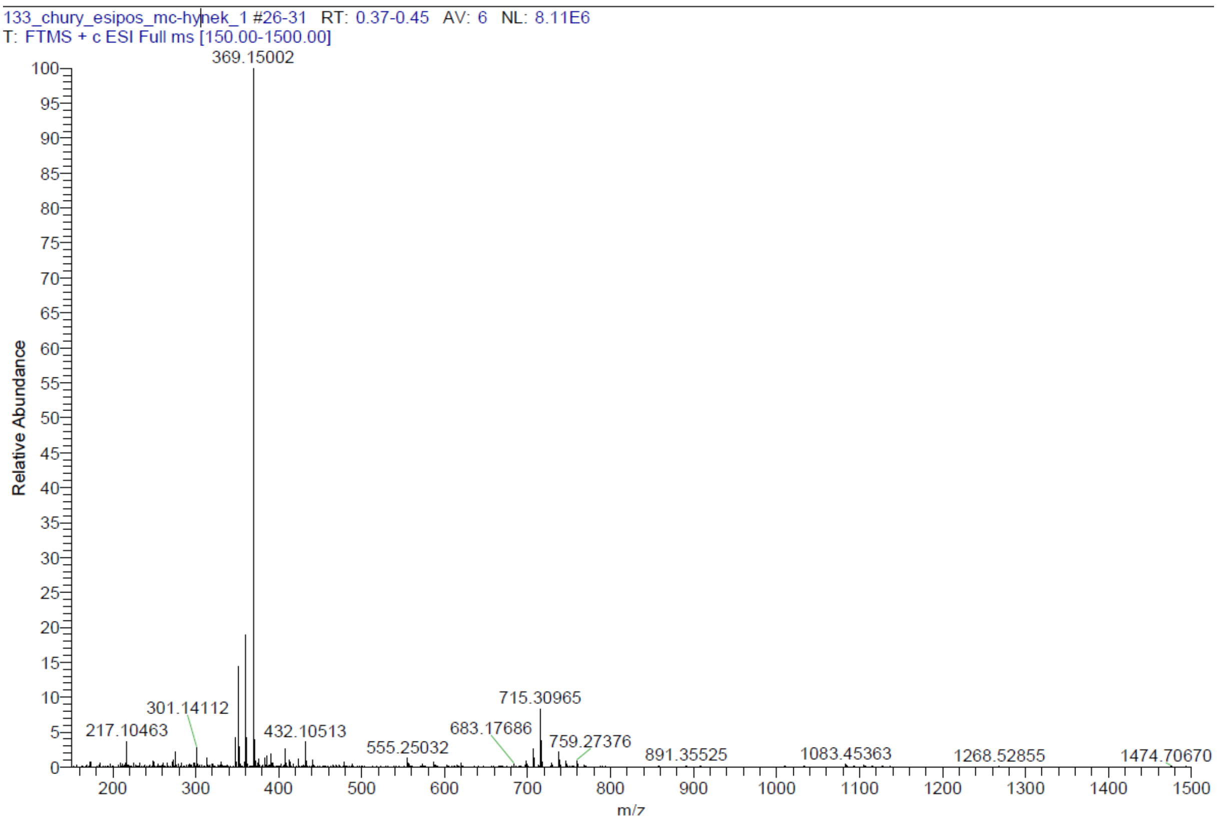

Figure S23: HRMS of compound **10** (ESI+)

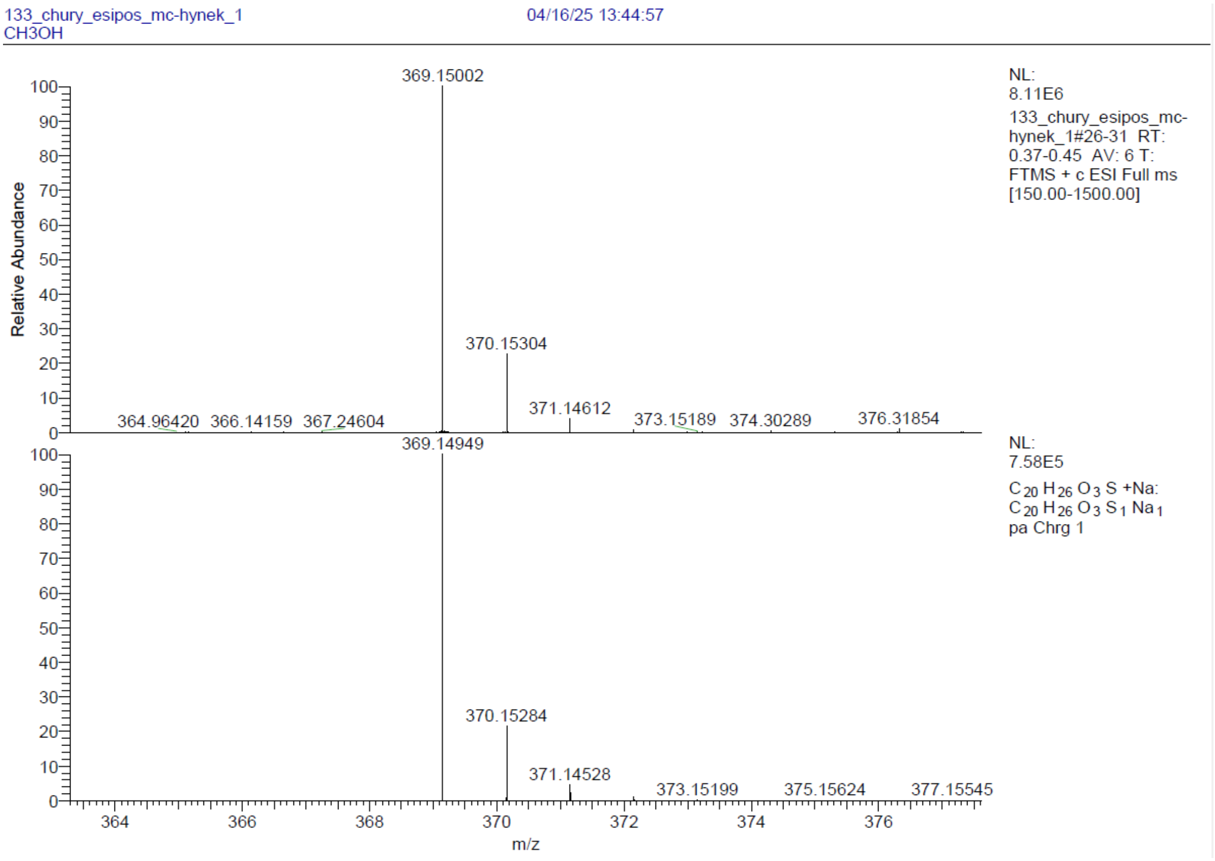

Figure S24: HRMS of compound **11** (ESI+) - isotopic distribution.

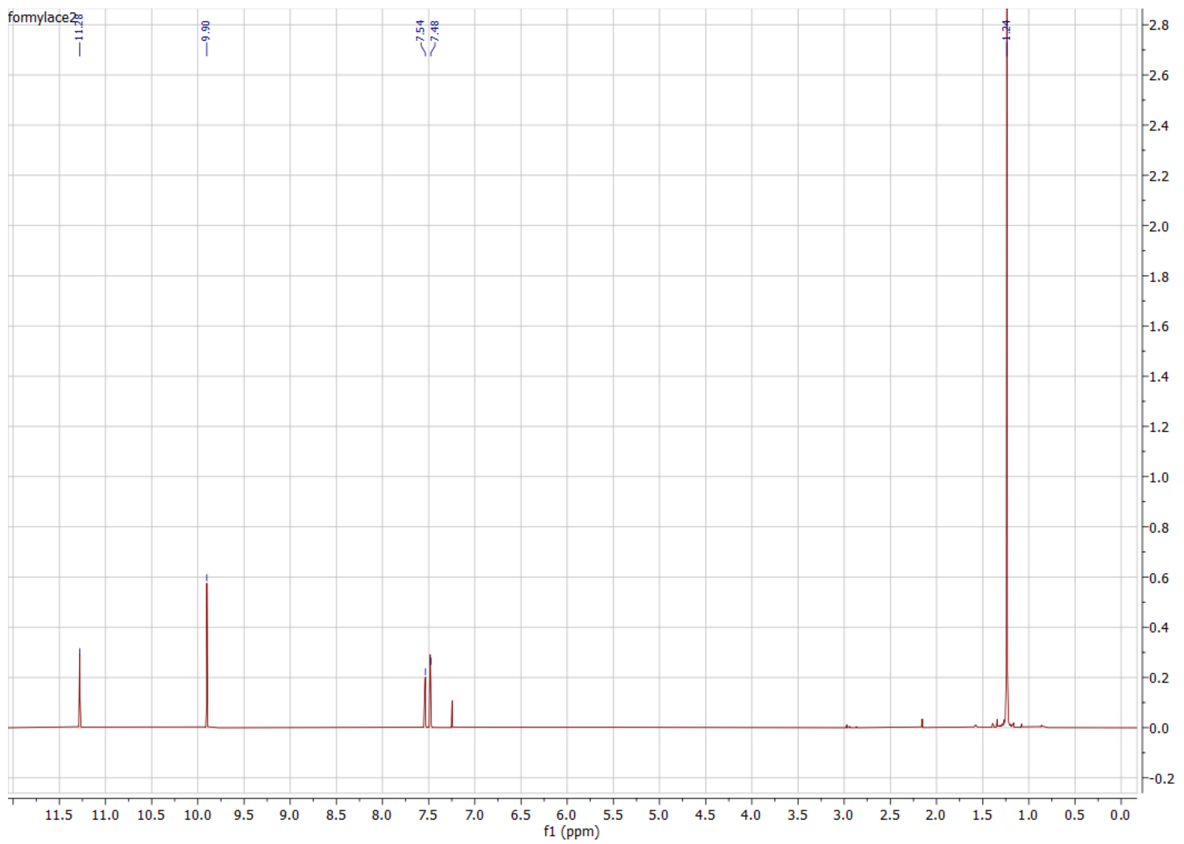

Figure S25: ^{1}H NMR of compound **11** ($CDCl_3$, 400 MHz, 298 K)

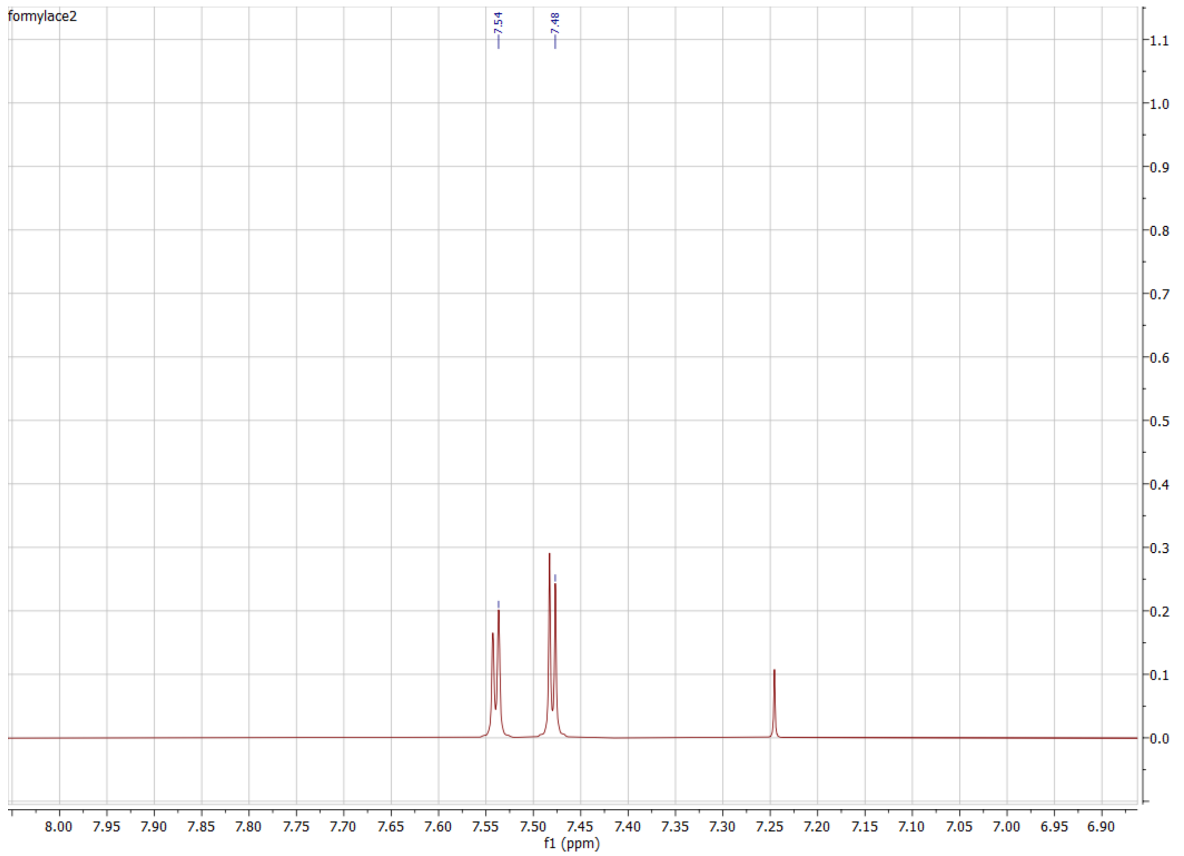

Figure S26: ^{1}H NMR of compound **11** – aromatic region ($CDCl_3$, 400 MHz, 298 K)

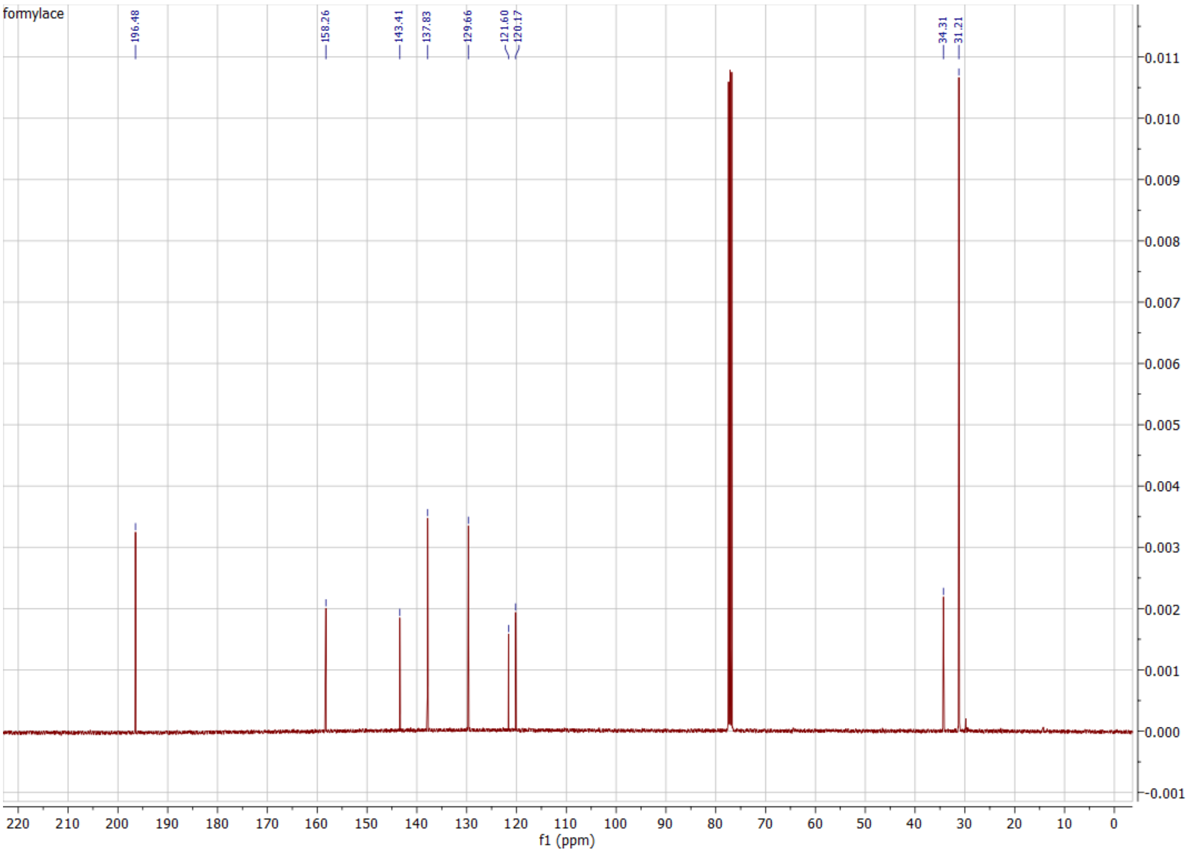

Figure S27: ^{13}C NMR of compound **11** ($CDCl_3$, 101 MHz, 298 K)

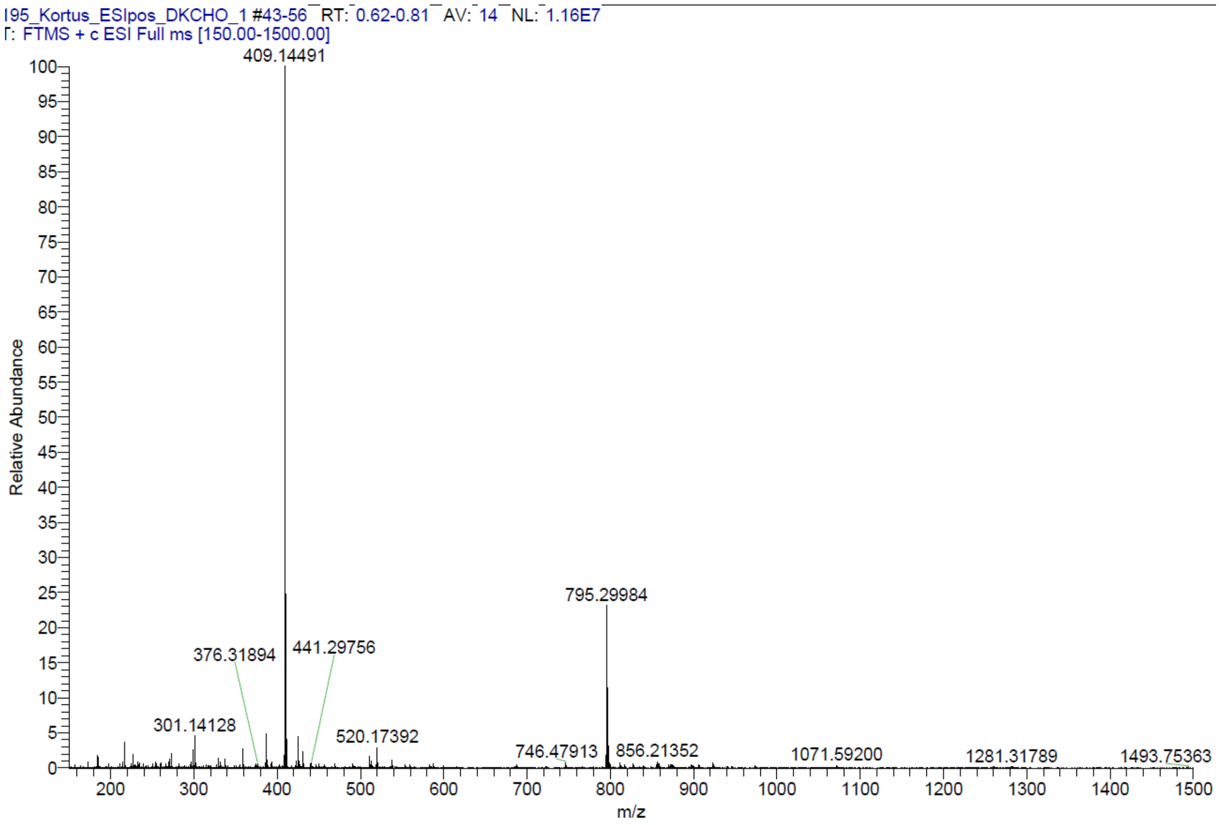

Figure S28: HRMS of compound **11** (ESI$^+$)

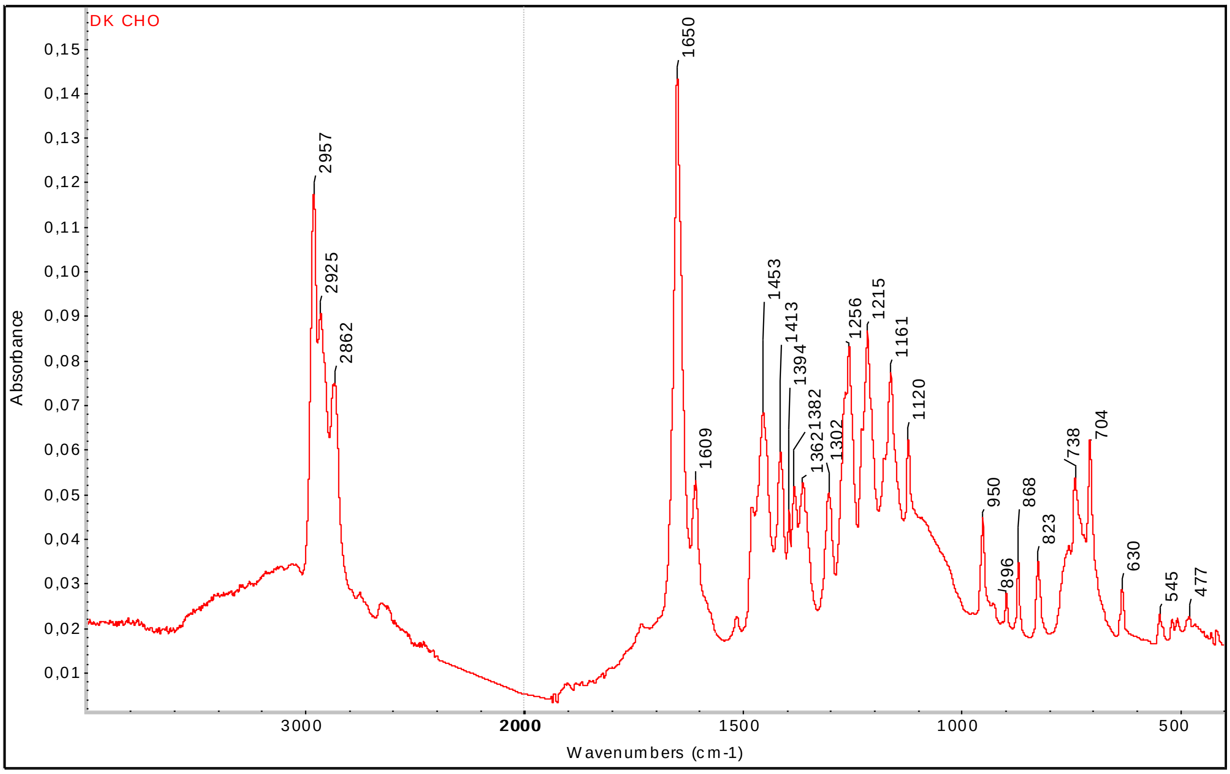

Figure S29: IR of compound **11** (ATR)

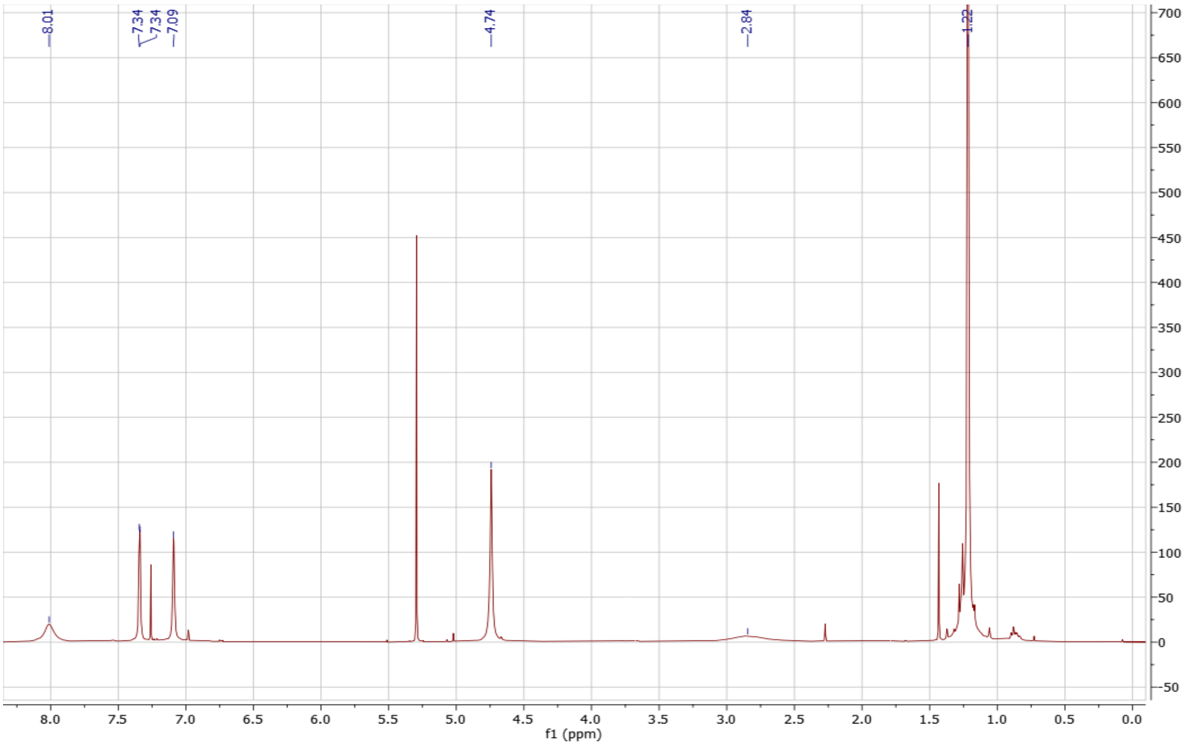

Figure S30: ^{1}H NMR of compound **12** ($CDCl_3$, 400 MHz, 298 K)

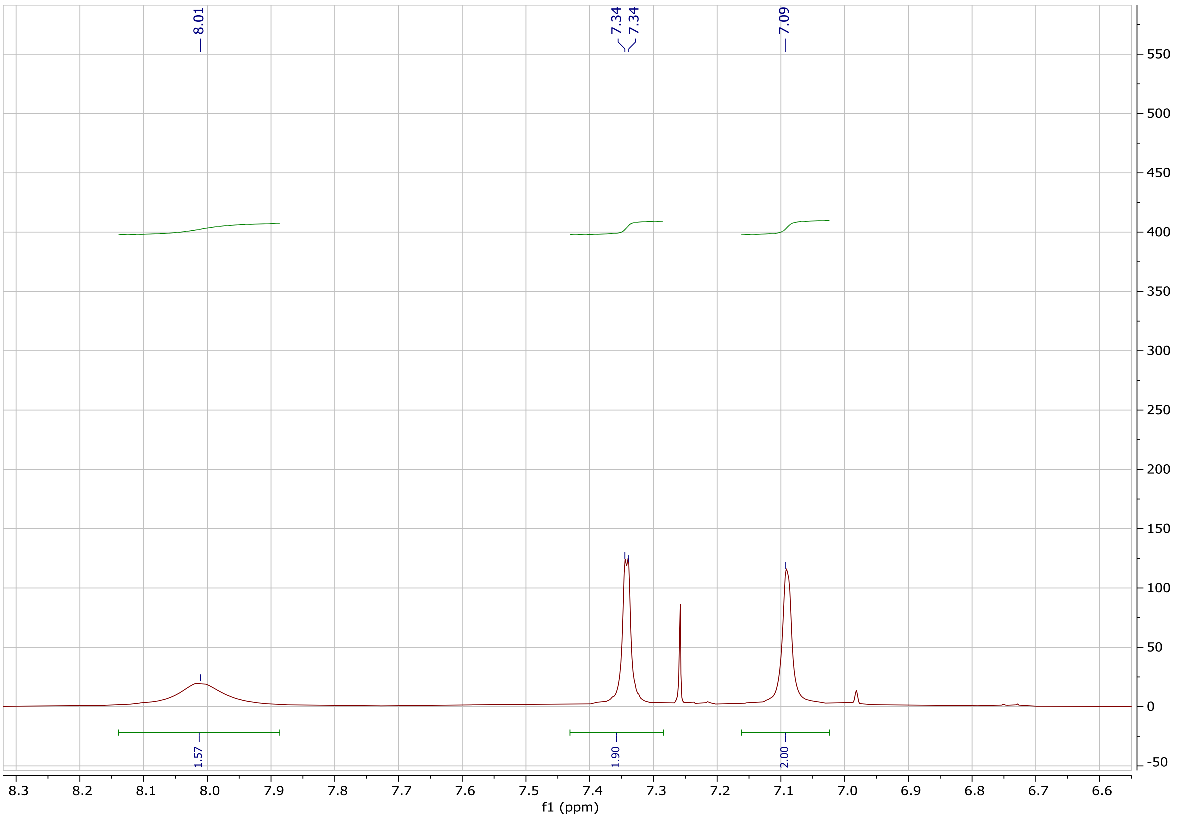

Figure S31: ^{1}H NMR of compound **12** – aromatic region ($CDCl_3$, 400 MHz, 298 K)

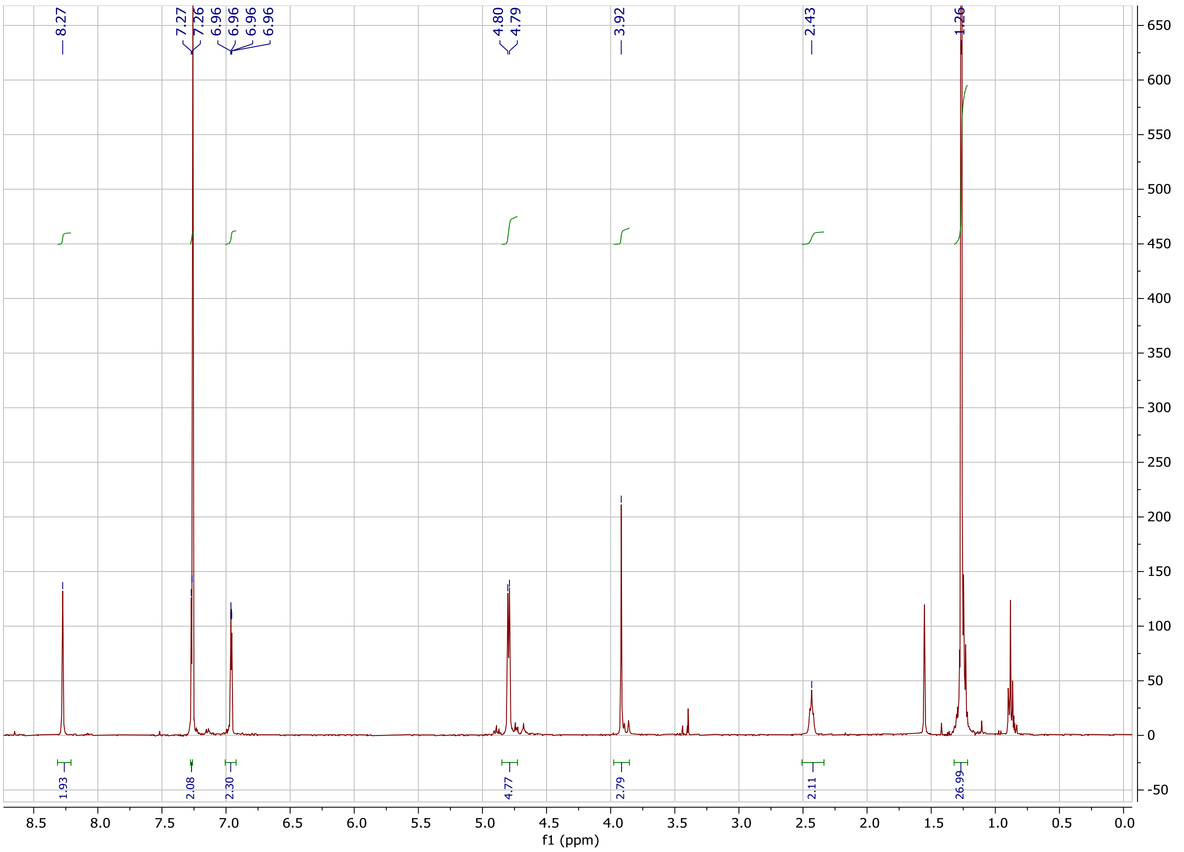

Figure S32: ^{1}H NMR of compound **13** ($CDCl_3$, 400 MHz, 298 K)

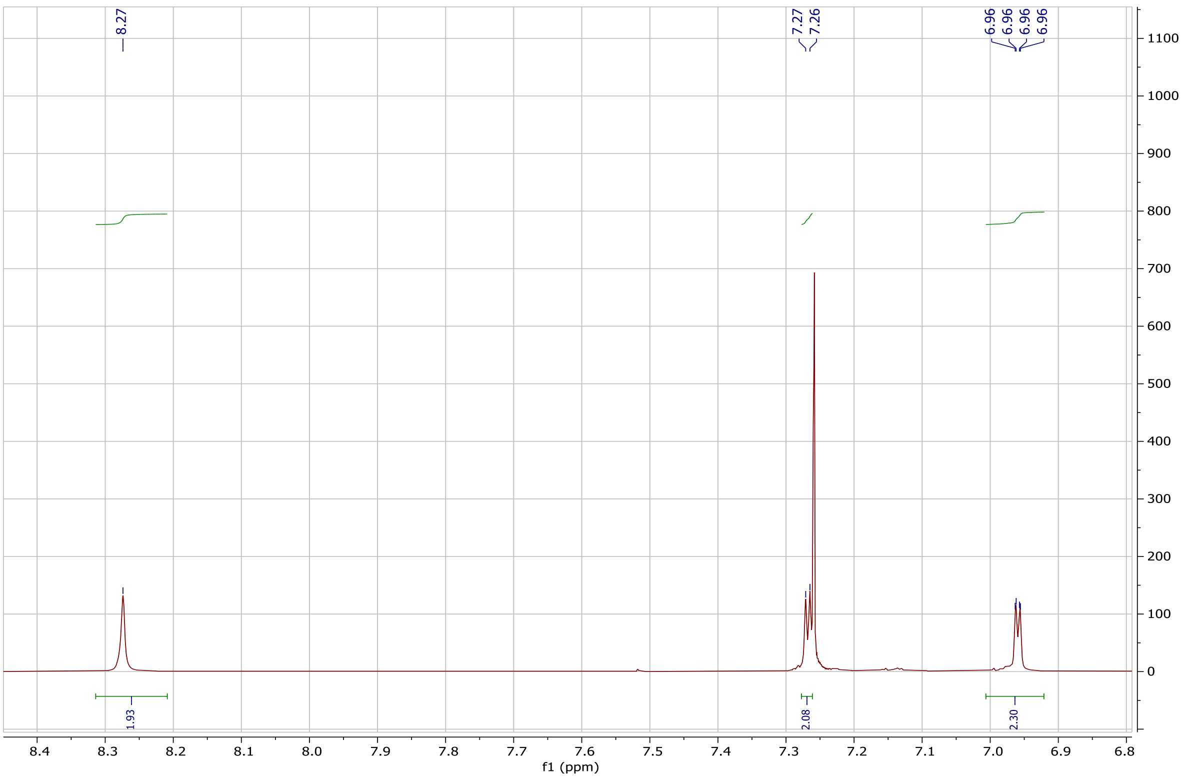

Figure S33: ^{1}H NMR of compound **13** – aromatic region ($CDCl_3$, 400 MHz, 298 K)

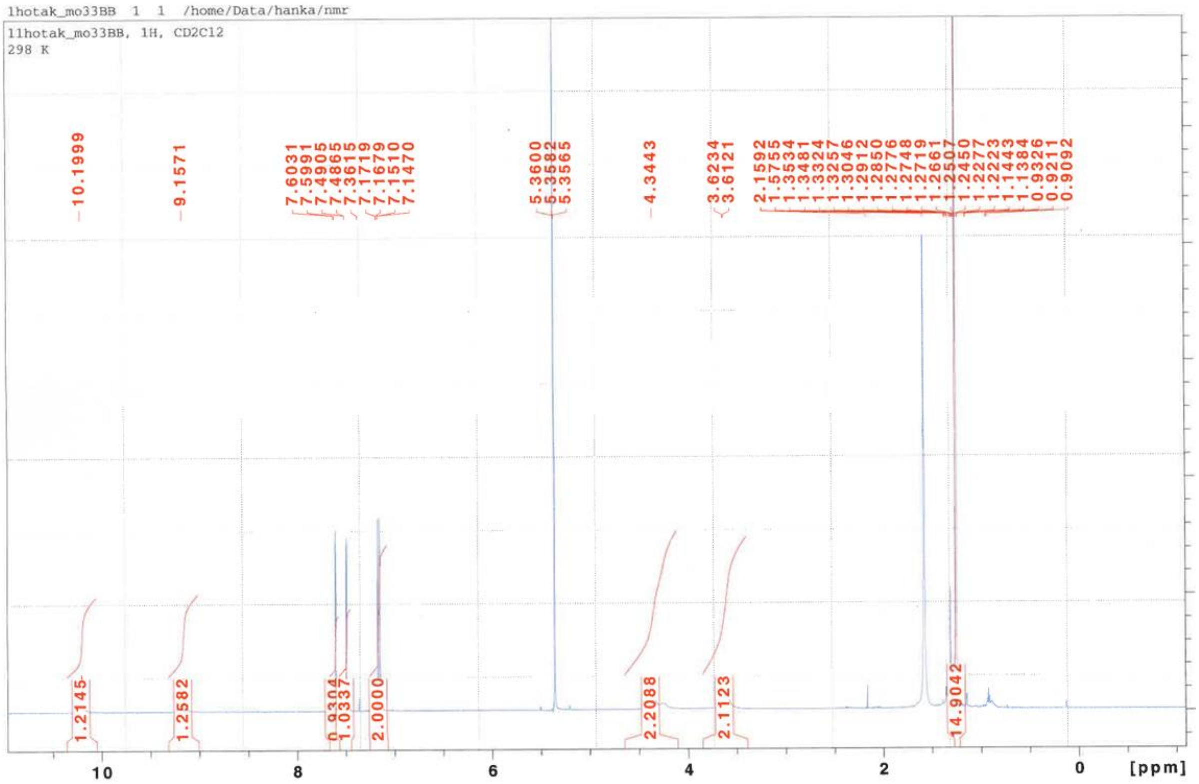

Figure S34: ^{1}H NMR of compound **14** – full spectrum (CD_2Cl_2, 600 MHz, 298 K)

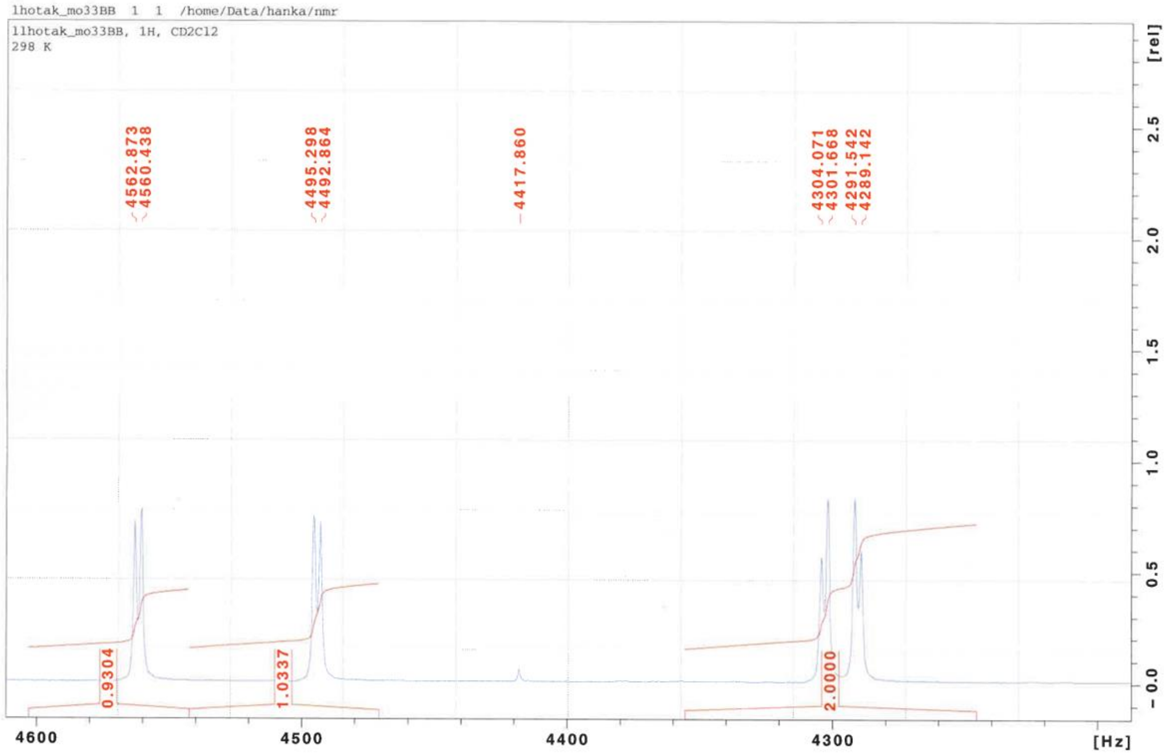

Figure S35: ^{1}H NMR of compound **14** – aromatic region (CD_2Cl_2, 600 MHz, 298 K)

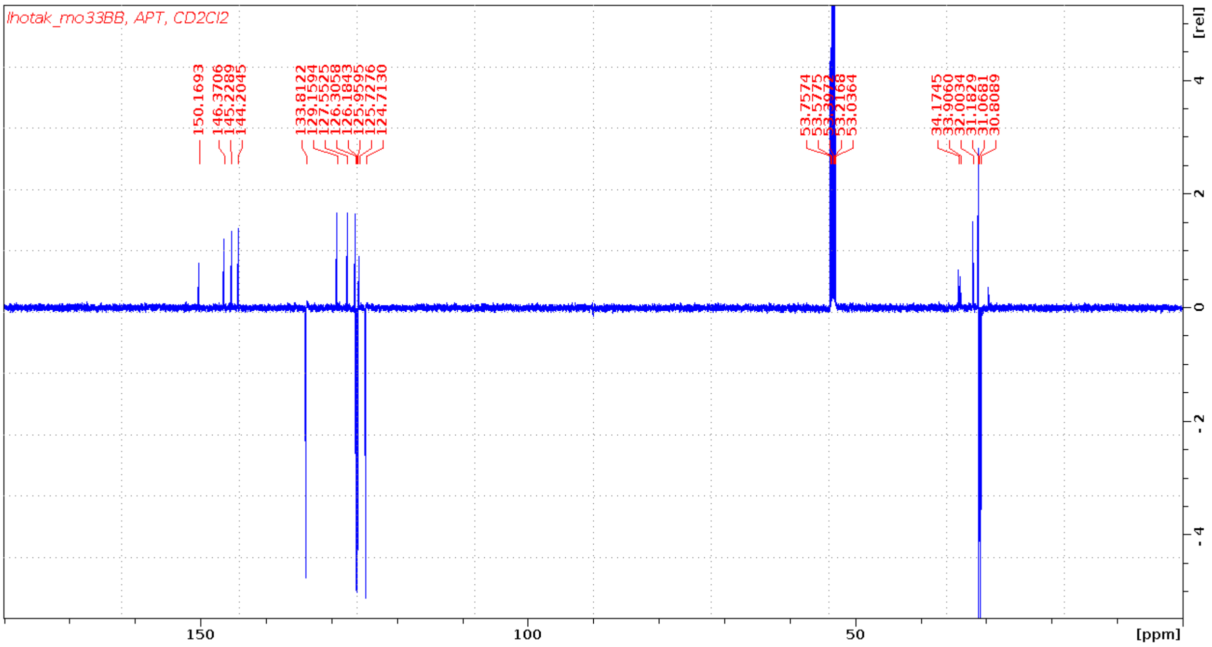

Figure S36: ^{13}C APT NMR of compound **14** – full spectrum (CD_2Cl_2, 150 MHz, 193 K)

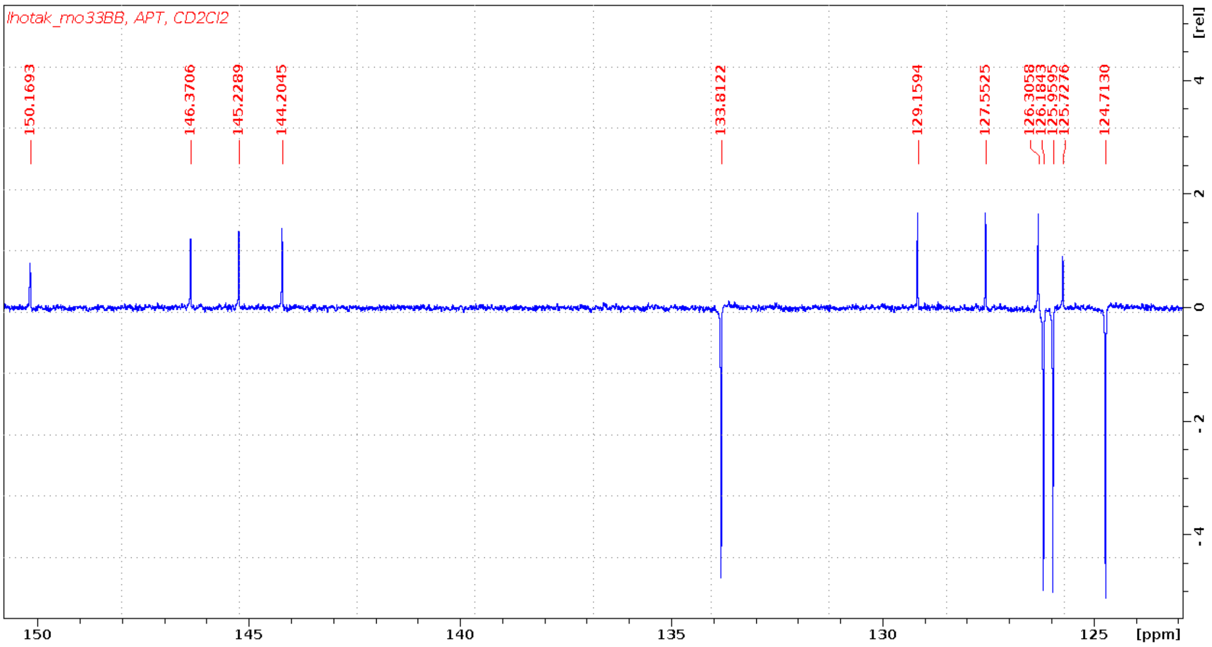

Figure S37: ^{13}C APT NMR of compound **14** – aromatic region (CD_2Cl_2, 150 MHz, 193 K)

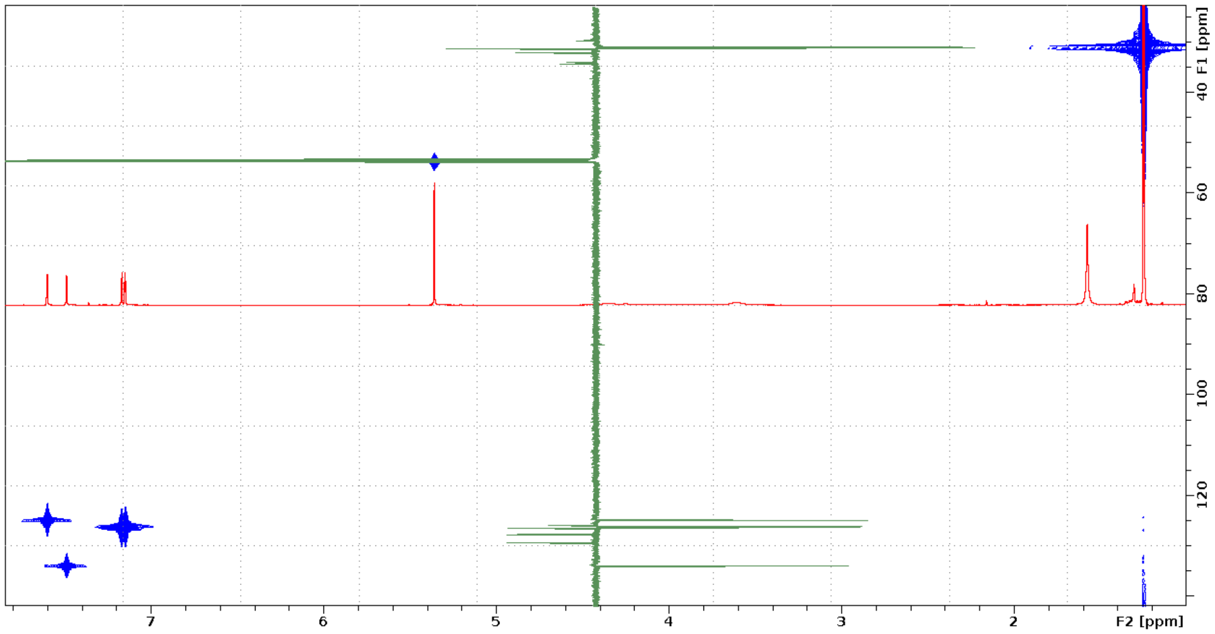

Figure S38: HMQC NMR of compound **14** (CD_2Cl_2 298 K)

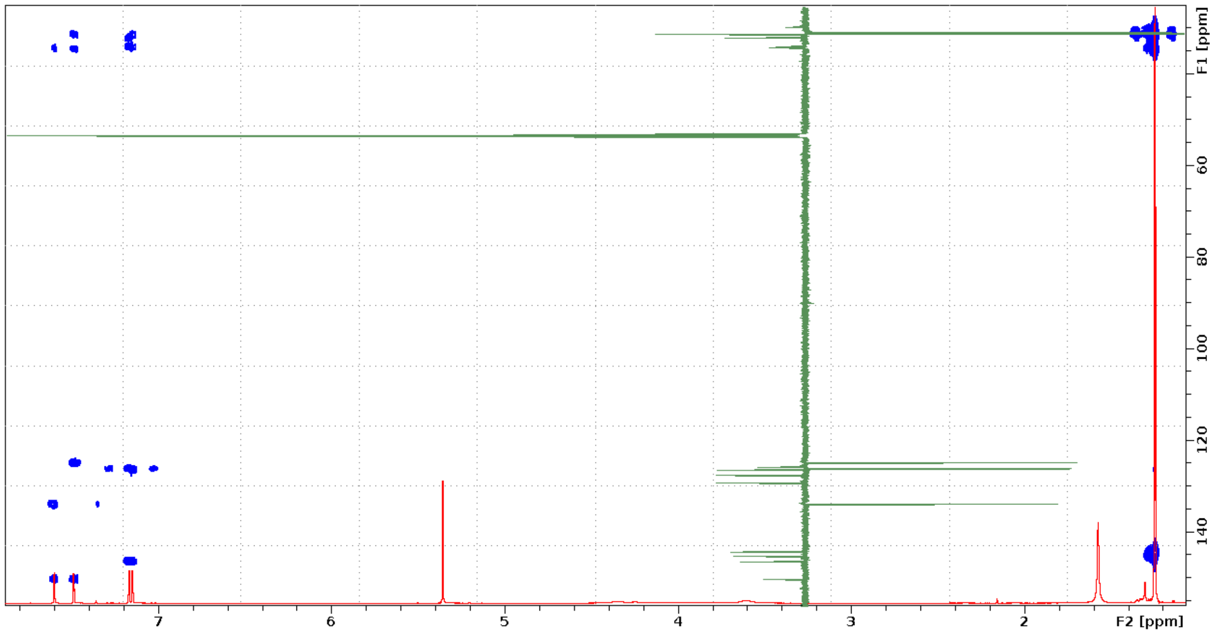

Figure S39: HMBC NMR of compound **14** – full spectrum (CD_2Cl_2 298 K)

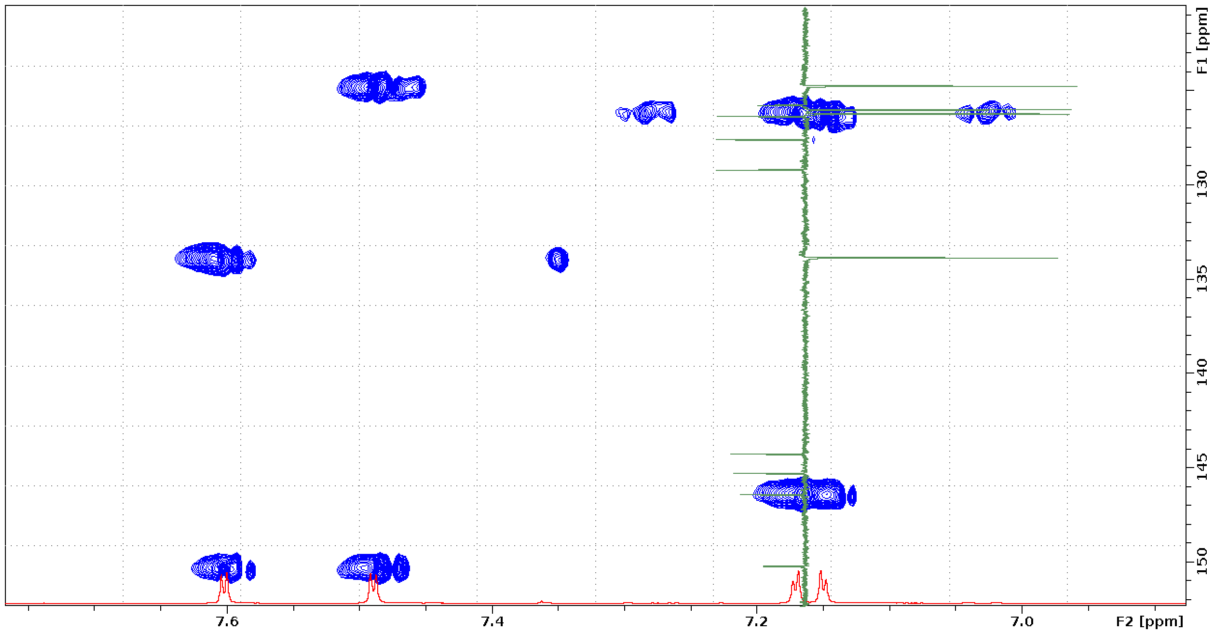

Figure S40: HMBC NMR of compound **14** – aromatic region (CD_2Cl_2 298 K)

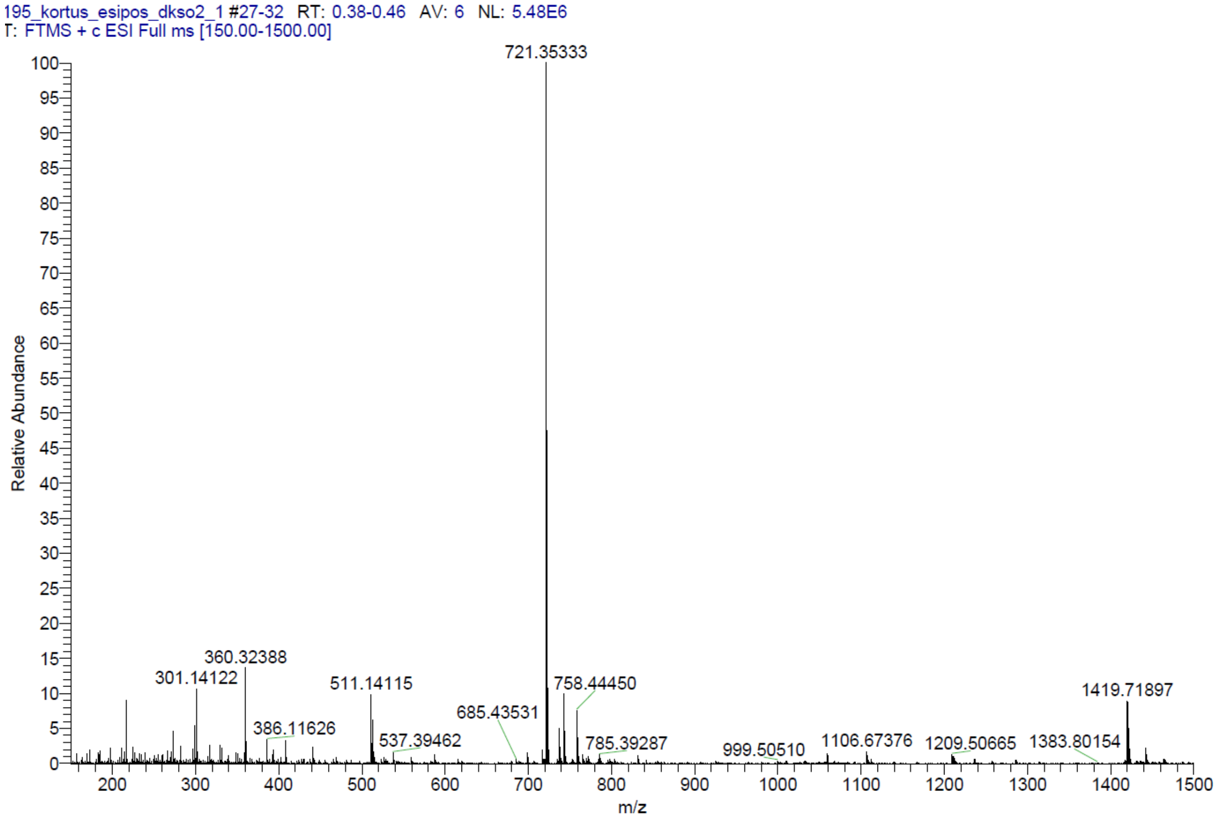

Figure S41: HRMS of compound **14** (ESI^+)

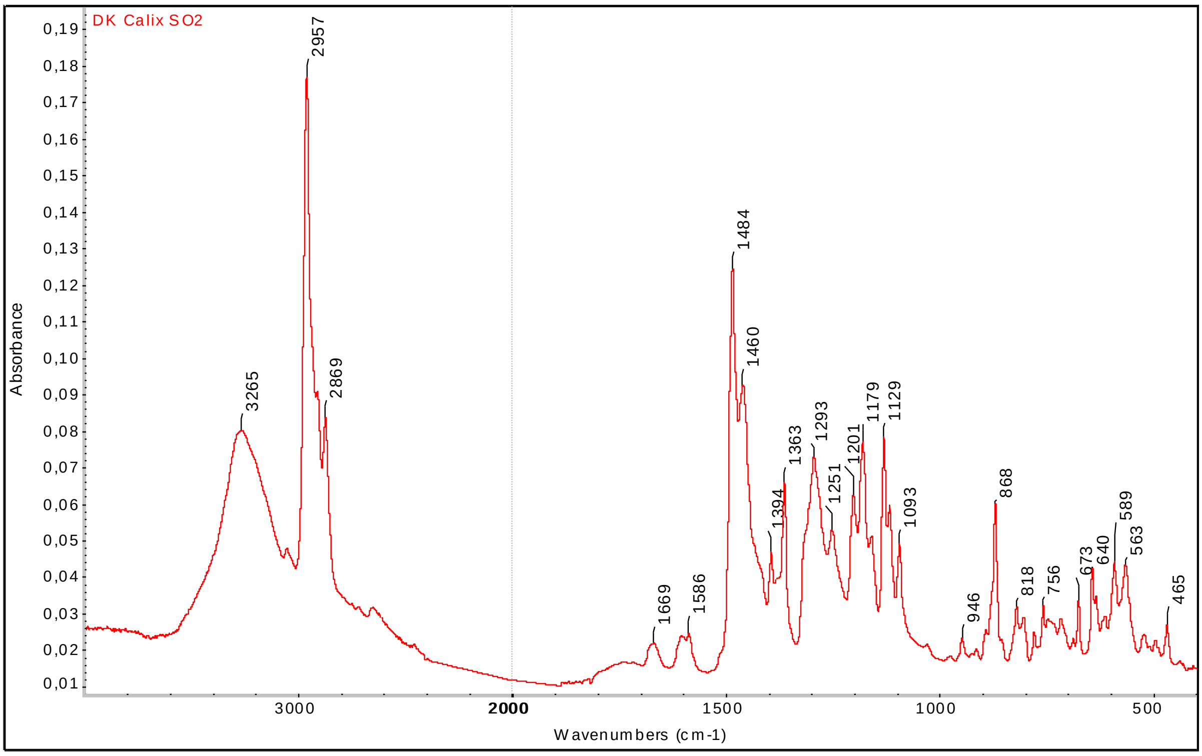

Figure S42: IR of compound **14** (KBr)

2. X-ray data

Crystallographic data for **14**.

$C_{43}H_{54}O_6S \cdot C_6H_{12}$, M = 783.12 g·mol^{-1}, tetragonal system, space group *P -4*, *a* = 12.7258(3) Å, *c* = 13.6553(5) Å, *Z* = 2, *V* = 2211.42(13) Å^3, D_c = 1.176 g·cm^{-3}, μ(Cu-Kα) = 1.017 mm^{-1}, crystal dimensions of 0.08 × 0.19 × 0.23 mm. Data were collected at 180 (2) K on a Bruker D8 Venture Photon II 7 diffractometer with Incoatec microfocus sealed tube Cu-Kα radiation. The data were integrated, scaled and corrected for absorption using Apex4. [1] The structure was solved by SIR92 [2] and anisotropically refined by full matrix least squares on *F* squared using the CRYSTALS [3] to final value of R_1 0.106 (3716 reflections) and *wR* = 0.261 (3930 independent reflections), ϑ_{max} = 69.7°, 417 refined parameters and 365 restrains. The hydrogen atoms bonded to carbon atoms were placed in calculated positions and refined with riding constrains. The hydrogen atoms bonded to oxygen atoms were not found, thus, they were placed to follow the shortest hydrogen bond 0.82 Å from the pivot oxygen atoms, further refinement was unstable. The disordered functional groups positions were found in difference electron density maps and refined with restrained geometry. Eventual minor deformations of the low-rim calix skeleton were neglected. DELU and SIMU restraints were used to keep overlapping ADP's acceptable. MCE [4] was used for visualization of electron density maps. The occupancy of disordered functional groups was initially refined constrained to full, during final stages it was refined fixed at 0.25 {sulfonyl), 0.5 (*tert*-butyl phenol) and 0.75 (methylene). It was extremely difficult to obtain single crystal suitable for X-ray data collection from experiments usually resulting in subtle polycrystalline aggregates. The data collection of the best single-crystal, obtained from repeated crystal growth attempts, took five days. The structure was deposited into Cambridge Structural Database under number CCDC 2469735.

1. *Bruker (2021). APEX4, SAINT and SADABS. Bruker AXS Inc., Madison, Wisconsin, USA.*
2. Altomare, A.; Cascarano, G.; Giacovazzo, C.; Guagliardi, A.; Burla, M. C.; Polidori, G.; Camalli, M., SIRPOW.92 - a program for automatic solution of crystal structures by direct methods optimized for powder data. *J. Appl. Crystallogr.* **1994,** 27, (3), 435-436.
3. Betteridge, P.; Carruthers, J.; Cooper, R.; Prout, K.; Watkin, D., CRYSTALS version 12: software for guided crystal structure analysis. *J. Appl. Crystallogr.* **2003,** 36, (6), 1487.
4. Rohlicek, J.; Husak, M., MCE2005 - a new version of a program for fast interactive visualization of electron and similar density maps optimized for small molecules. *J. Appl. Crystallogr.* **2007,** 40, (3), 600-601.

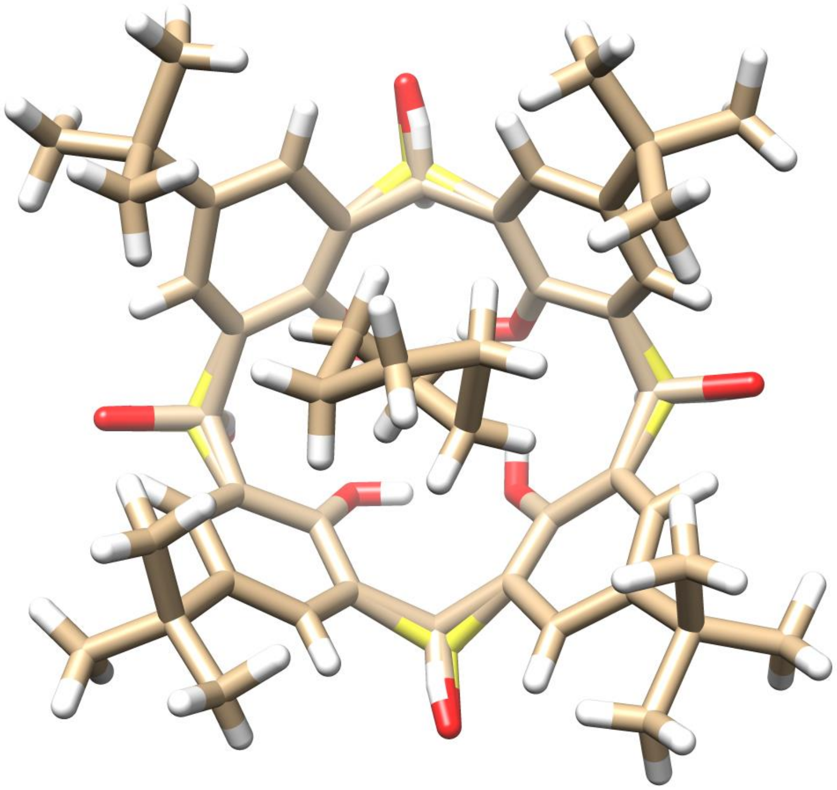

Figure S43: X-ray structure of compound **14** – complex with cyclohexane.

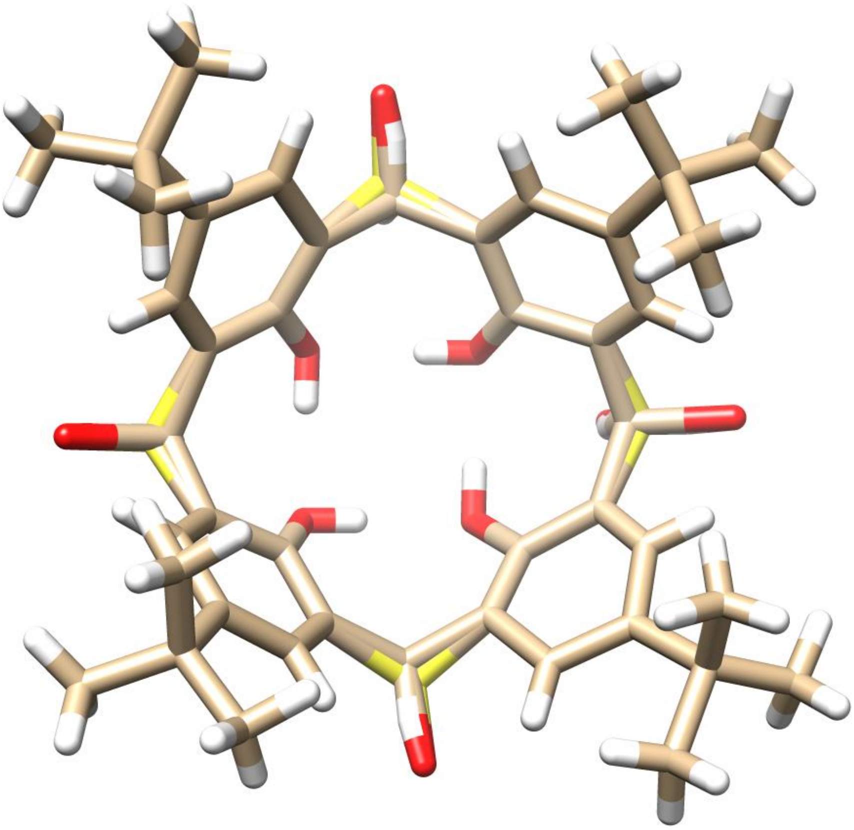

Figure S44: X-ray structure of compound **14** –cyclohexane omitted.

3. Variable Temperature NMR

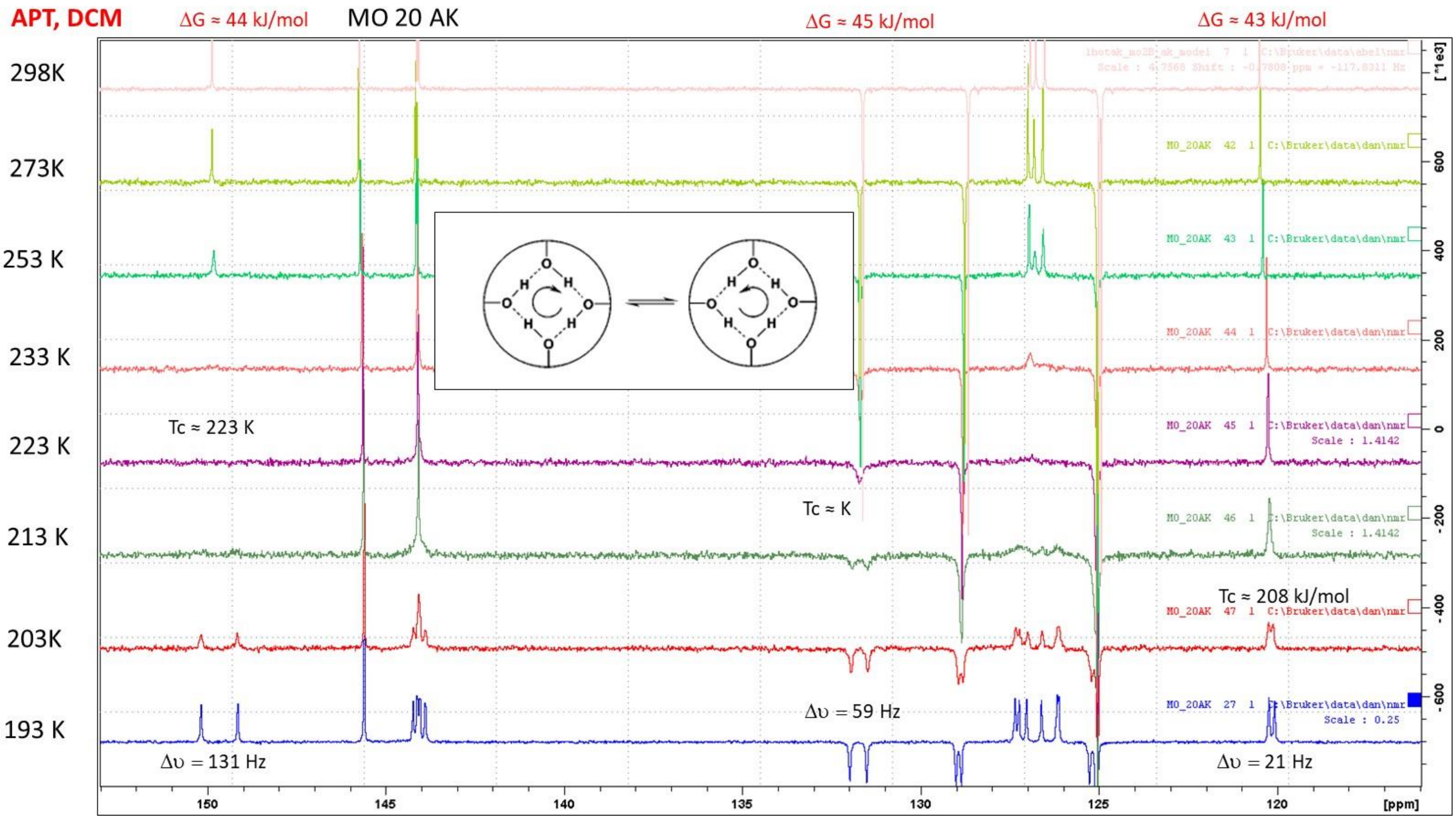

Figure S45: VT ^{13}C NMR of compound **7** (CD_2Cl_2, 150 MHz).

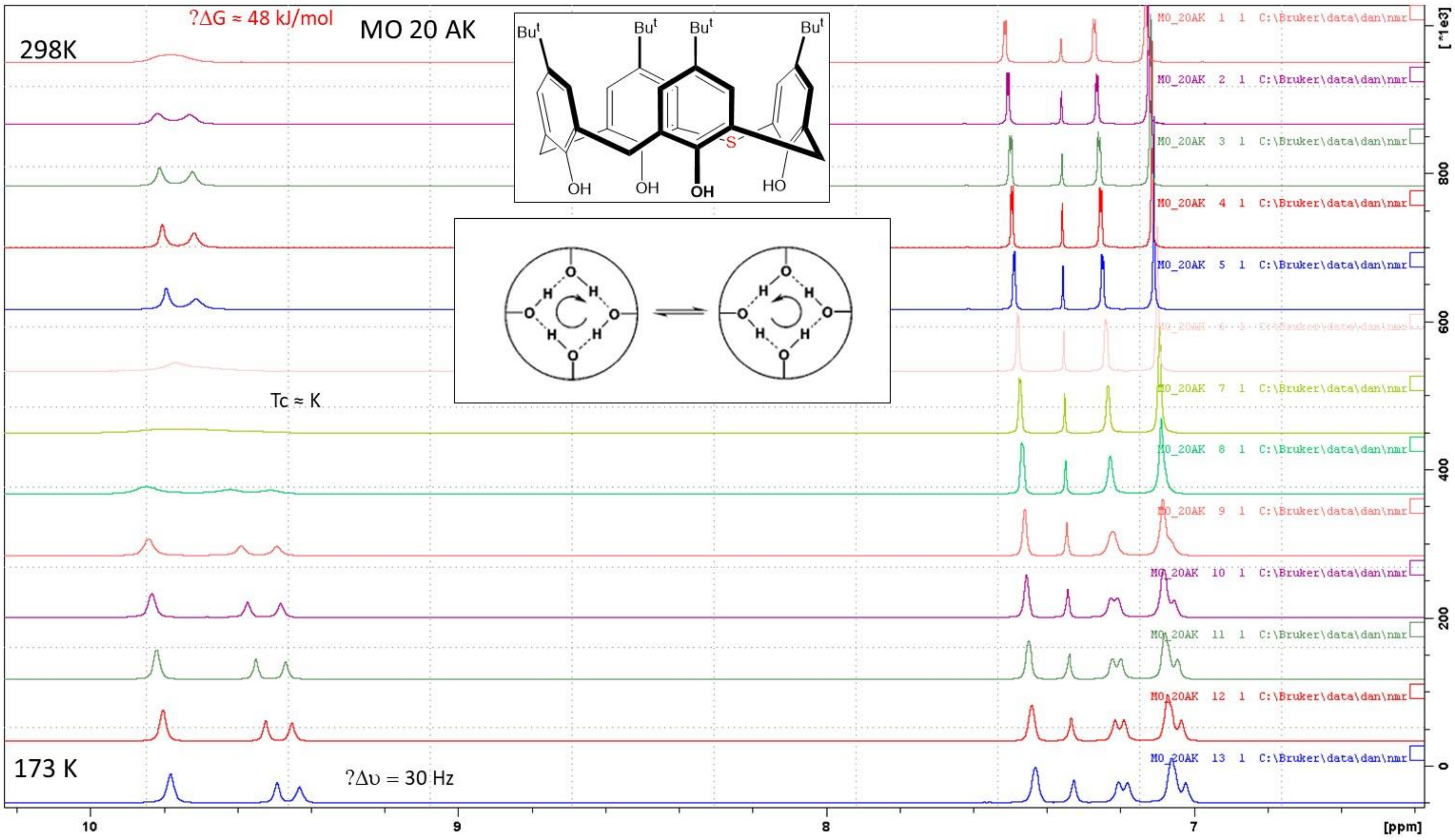

Figure S46: VT ^{1}H NMR of compound **7** (CD_2Cl_2, 600 MHz).

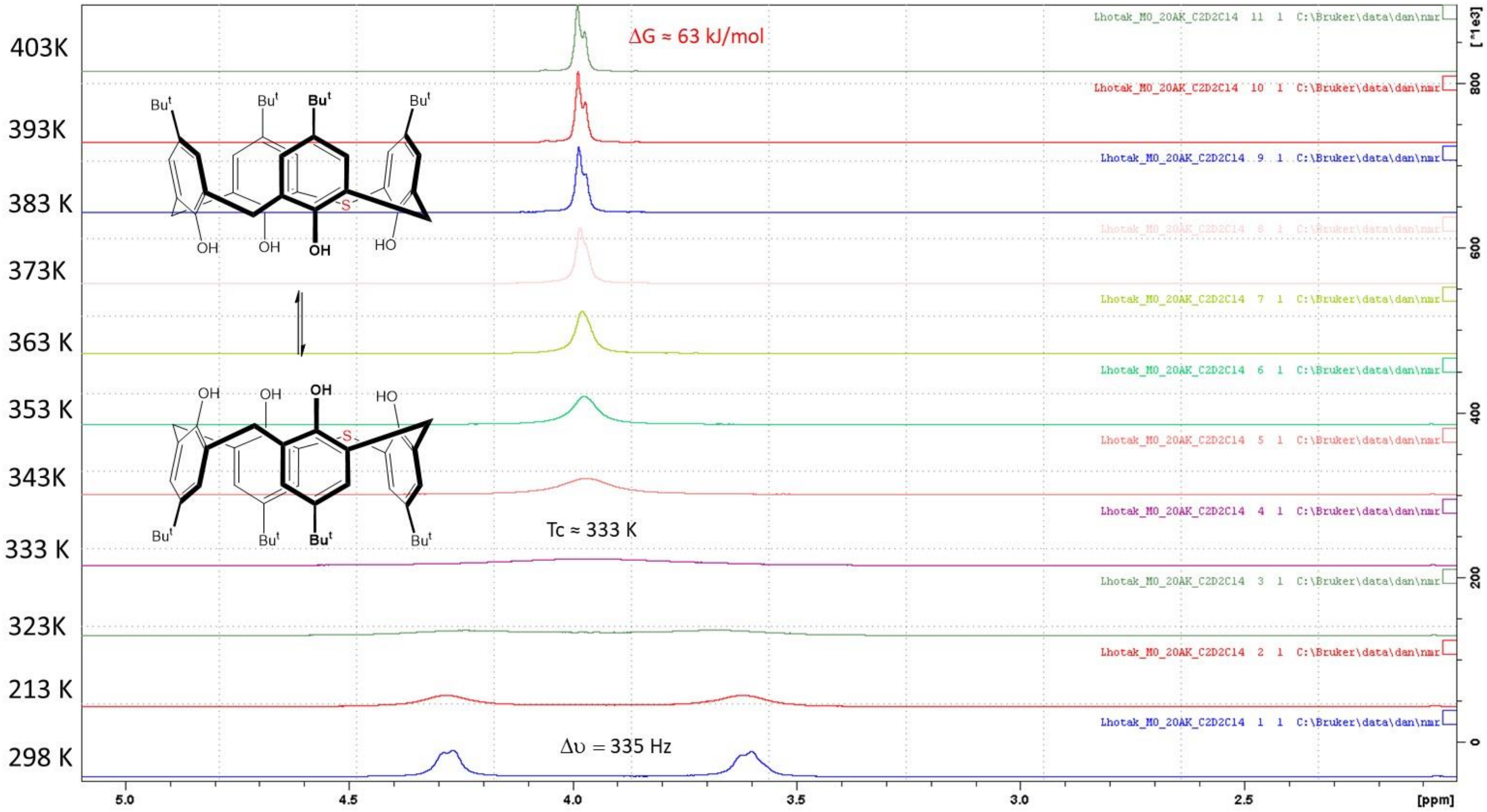

Figure S47: VT ^{1}H NMR of compound **7** ($CDCl_2$-$CDCl_2$, 600 MHz).

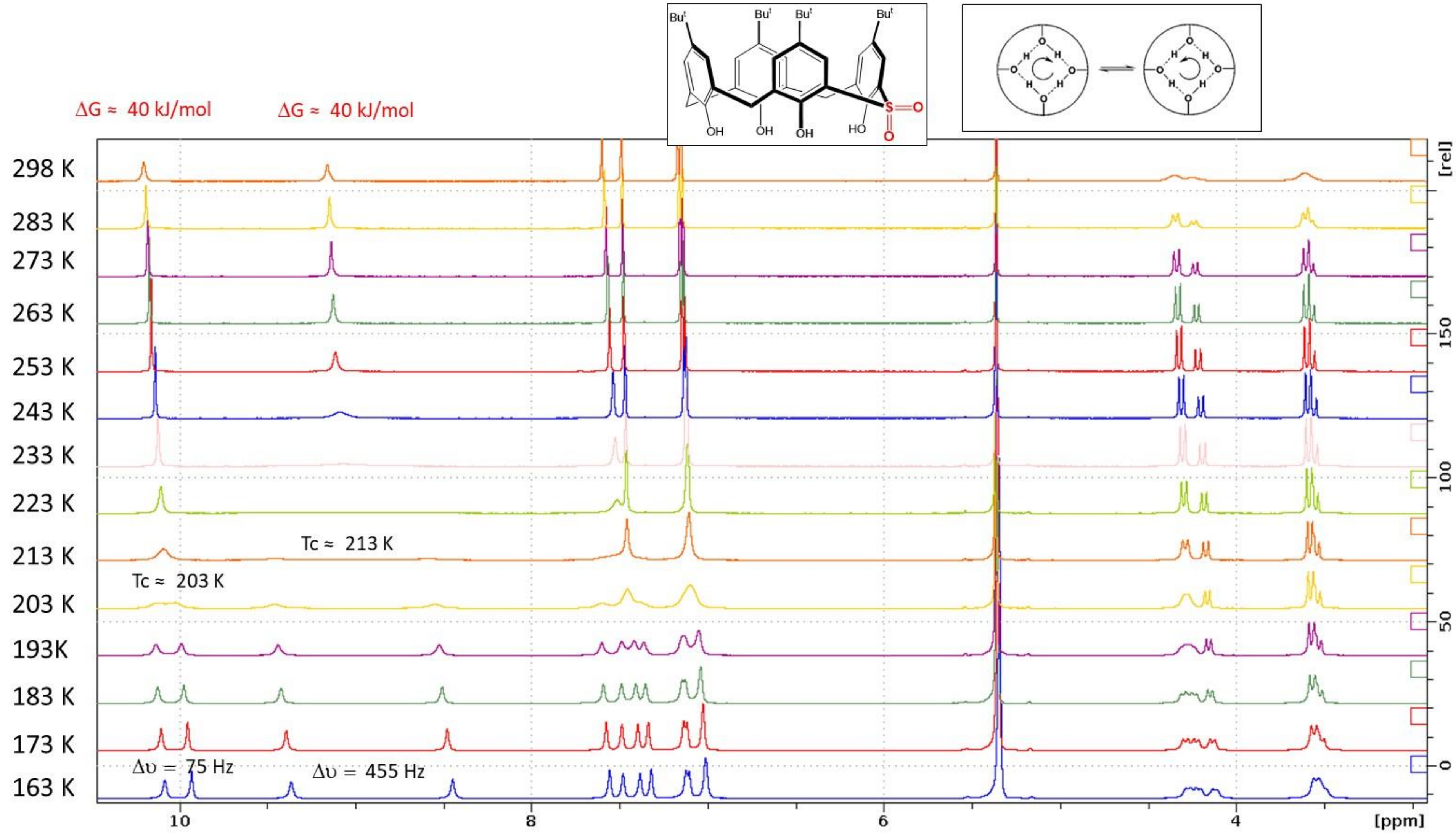

Figure S48: VT ^{1}H NMR of compound **14** (CD_2Cl_2, 150 MHz).

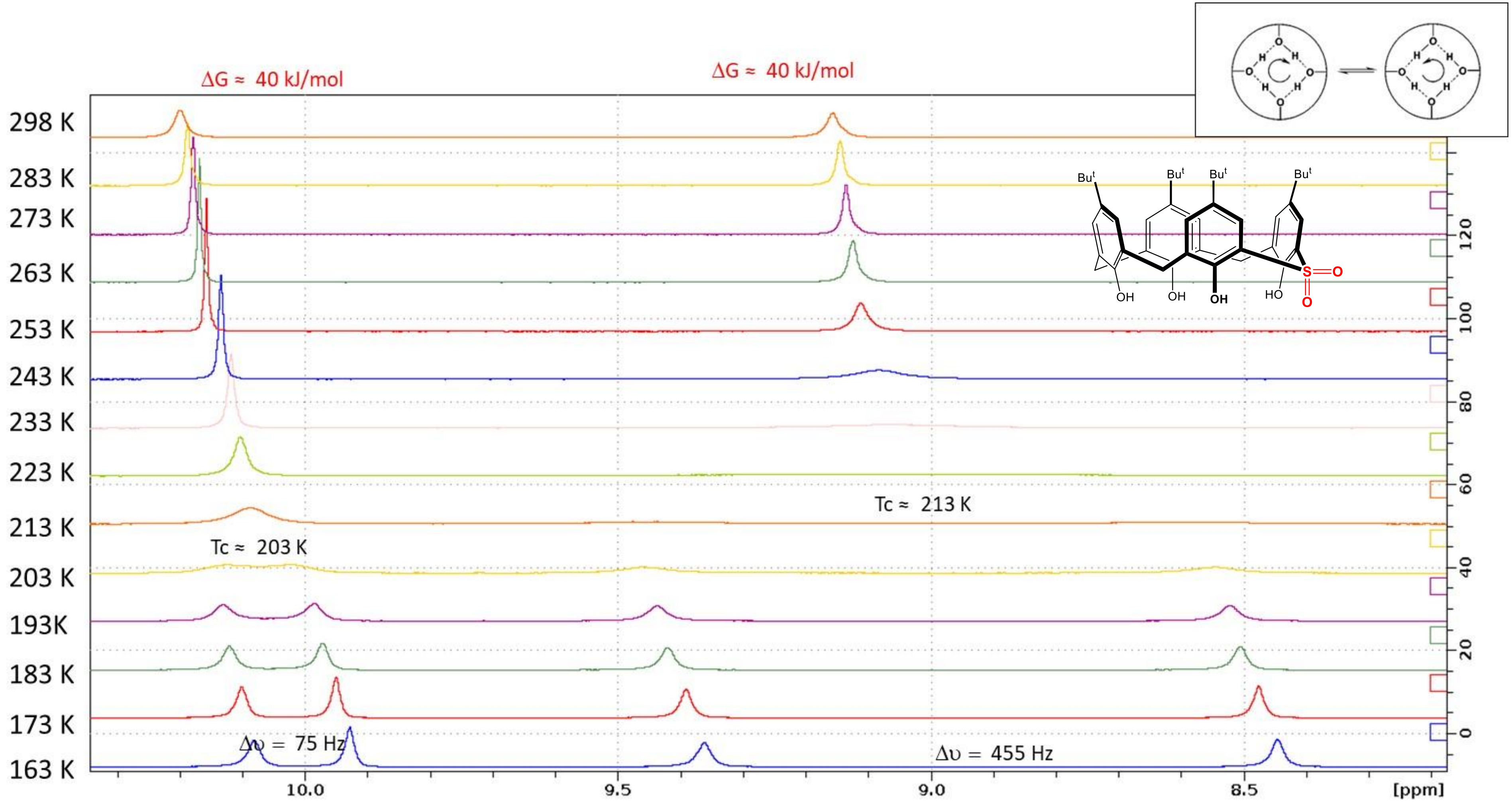

Figure S49: VT ^{1}H NMR of compound **14** – phenolic signals (CD_2Cl_2, 150 MHz).

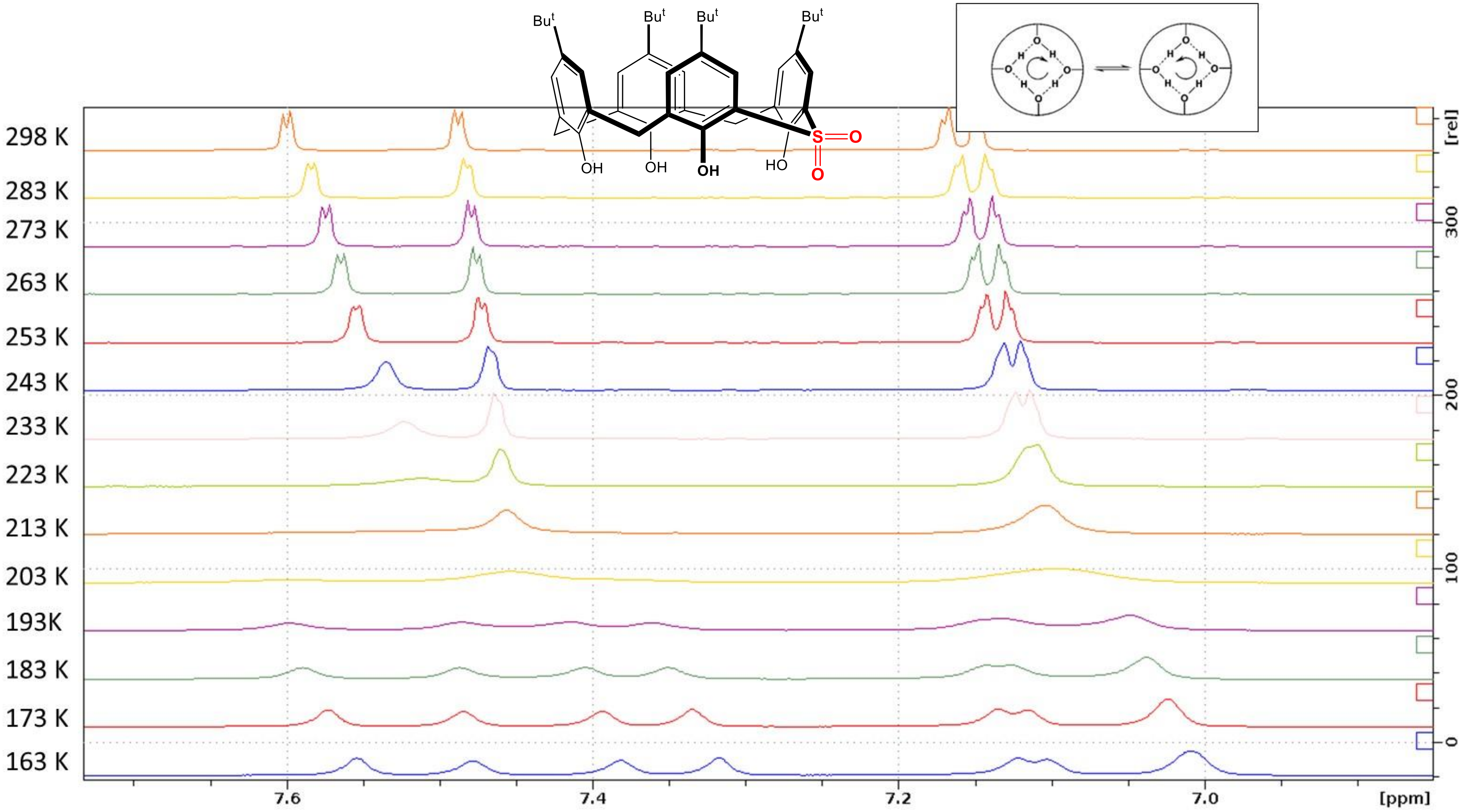

Figure S50: VT ^{1}H NMR of compound **14** – aromatic part (CD_2Cl_2, 150 MHz).

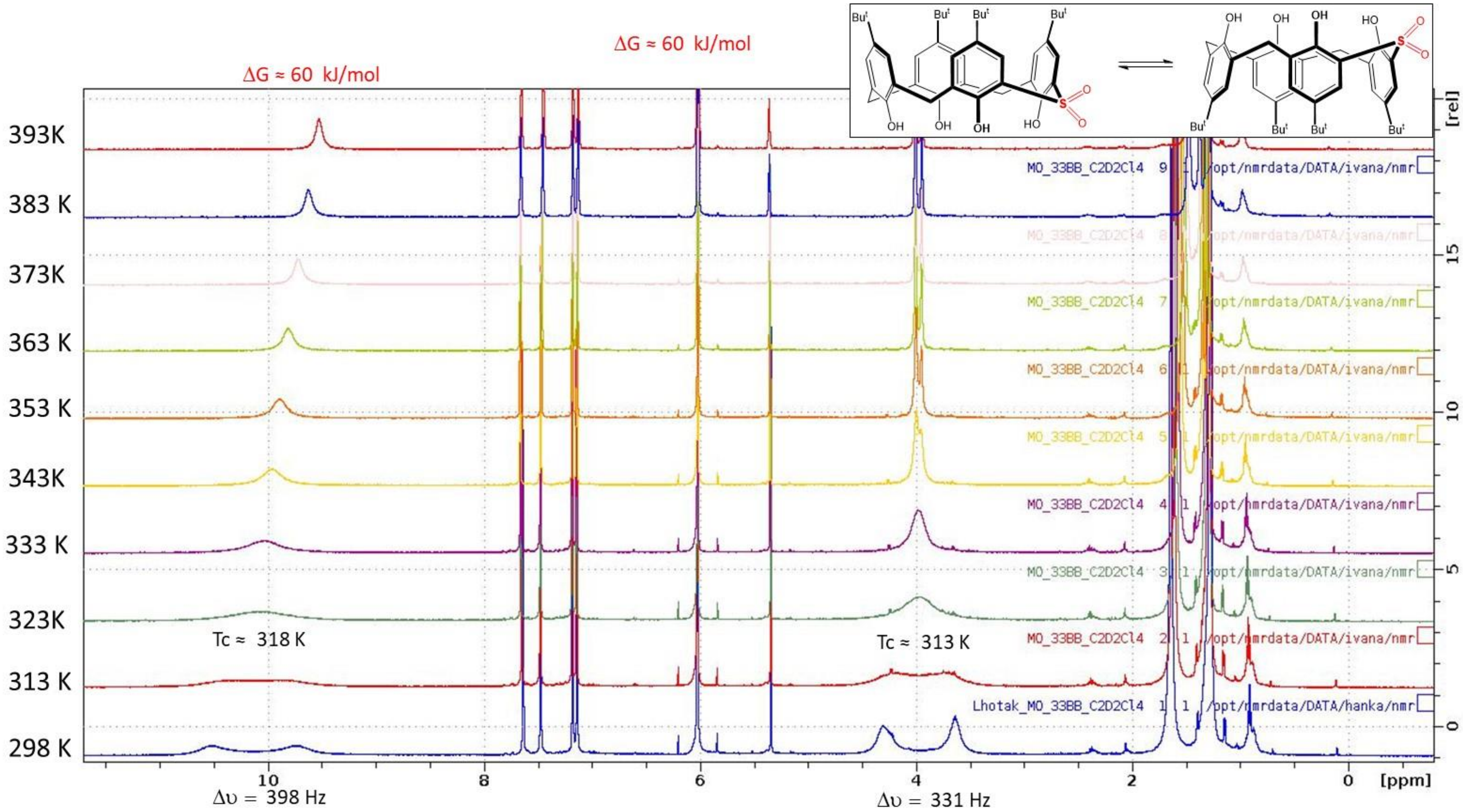

Figure S51: VT ^{1}H NMR of compound **14** ($CDCl_2$-$CDCl_2$, 600 MHz).
